# Study of Mathematical Models Describing the Thermal Decomposition of Polymers Using Numerical Methods

**DOI:** 10.3390/polym17091197

**Published:** 2025-04-27

**Authors:** Gaziza M. Zhumanazarova, Akmaral Zh. Sarsenbekova, Lyazzat K. Abulyaissova, Irina V. Figurinene, Rymgul K. Zhaslan, Almagul S. Makhmutova, Raissa K. Sotchenko, Gulzat M. Aikynbayeva, Jakub Hranicek

**Affiliations:** 1 Department of Chemical Technology and Ecology, Karaganda Industrial University, Temirtau 101400, Kazakhstan; gaziza.zhumanazarova@mail.ru (G.M.Z.);; 2 Chemistry Faculty, Karaganda Buketov University, Karaganda 100024, Kazakhstan; 3 School of Pharmacy, Karaganda Medical University, Karaganda 100024, Kazakhstan; electrochimik@mail.ru (I.V.F.);; 4 Department of Analytical Chemistry, Charles University, 128 43 Praha, Czech Republic

**Keywords:** matrix, polymer, polypropylene glycol fumarate phthalate, polymer degradation, thermodynamic characteristics

## Abstract

This research presents the results of a combined numerical and experimental study of the thermal decomposition behavior of copolymers based on polypropylene glycol fumarate phthalate. The thermal decomposition of polymers plays a key role in various fields, such as waste recycling and energy recovery, and in the development of new materials. The objective of this study is to model the degradation kinetics using thermogravimetric data, matrix-based numerical methods, and quantum chemical calculations. To solve the resulting systems of linear algebraic equations (SLAEs), matrix decomposition algorithms (QR, SVD, and Cholesky) were employed, which enabled the determination of activation energy values for the process. Comparison of the activation energy (*E_a_*) results obtained using the decomposition method of Cholesky (207.21 kJ/mol), normal equations (205.22 kJ/mol), singular value decomposition (206.23 kJ/mol), and QR decomposition (206.23 kJ/mol) showed minor changes that were associated with the features of each method. Quantum chemical calculations based on density functional theory (DFT) at the B3LYP/6-31G(*d*) level were performed to analyze the molecular structure and interpret the IR spectra. This study establishes that the content of functional groups (ether and ester) and the type of chemical bonds exert critical influences on the decomposition mechanism and associated thermal parameters. The results confirm that the polymer’s structural architecture governs its thermal stability. The scientific novelty of this work lies in the integration of numerical approximation methods and quantum chemical analysis for investigating the thermal behavior of polymers. This approach is applied for the first time to copolymers of this composition and may be employed in the design of heat-resistant materials for agricultural and environmental applications.

## 1. Introduction

Modern polymers are essential in industries such as packaging, construction, electronics, and medicine [1]. Special attention is given to copolymers based on polypropylene glycol fumarate phthalate [2]. While these polymers are widely used, their thermal decomposition mechanisms and kinetic features remain poorly understood. To gain a deeper understanding of these processes, it is crucial to employ computational methods. These methods enable the modeling of polymer decomposition kinetics and prediction of their stability under various temperature conditions.

As interest in “smart” polymers—specifically those based on unsaturated polyester resins (UPRs)—continues to grow, it has become necessary to conduct more detailed studies of their behavior. These polymers are noteworthy for their ability to exhibit significant changes in conformation in response to even slight fluctuations in external factors. Currently, polymers based on unsaturated polyester resins are often obtained by radical polymerization. A pioneering study by Benig [3,4] first described the interaction of unsaturated polyester resins with vinyl monomers, opening new possibilities for copolymerization.

Initially, unsaturated polyesters were found to copolymerize with vinyl acetate, styrene, and methyl methacrylate [5]. This discovery led to more extensive research covering a wider array of monomers, including both simple [6] and complex allyl ethers [7], vinyl formates [8], and others.

The pioneers in the field of studying the reaction kinetics of copolymerization of unsaturated polyesters under elevated temperature conditions are E.G. Zilberman and L.N. Sedov [9]. In comparing the results obtained using chemical methods, modified dilatometry, and extraction analysis, the aforementioned researchers found a direct relationship between the intensity of the reaction (with a consistent increase in the slope of the curves) and the temperature rise, which can be characterized by a specific value [10].

The literature extensively covers the study of the thermal properties of copolymers of unsaturated polyester resins with vinyl monomers [11]. However, similar studies on copolymers of unsaturated polyester resins with ionogenic monomers are lacking, although this opens up significant opportunities for the synthesis of new copolymers [12].

Extensive research has been conducted on methods for producing unsaturated polyester resins [12,13], including one of the key raw materials—polypropylene glycol fumarate phthalate. This raw material is used to produce the proposed copolymers, which can be applied as hydrogels in the cultivation of vegetable crops. It is worth noting that the technology for producing this raw material has been fully developed [14,15].

In addition to their application in agriculture as moisture-retaining agents, hydrogels based on unsaturated polyester resins are also considered promising materials for wastewater treatment. Due to their porous structure, high sorption capacity, and potential for chemical modification, these materials can be effectively utilized for the removal of heavy metals, organic dyes, and other pollutants from industrial and municipal effluents. One of the most efficient and environmentally friendly approaches is photocatalytic water treatment, in which the degradation of contaminants occurs under light irradiation in the presence of photosensitive nanomaterials. Hydrogels can be functionalized with such photocatalysts (e.g., TiO_2_, ZnO, or CuAlS_2_), enabling the activation of photodegradation processes of organic compounds under ultraviolet or solar radiation. Importantly, photocatalytic water purification is widely used for the degradation of organic dyes, which are commonly found in the wastewater of the textile, leather, food, and pharmaceutical industries. These compounds are resistant to natural biodegradation, making photocatalysis one of the most effective and eco-friendly methods for their removal. This makes the described copolymers attractive not only for agricultural but also for environmental applications, opening new avenues for their practical implementation.

In the context of developing efficient photocatalytic systems for wastewater treatment, the study by Kadam et al. [16]. is of particular interest. The authors [16] investigated the photocatalytic degradation of Rose Bengal dye using zinc oxide (ZnO) nanoparticles and molybdenum-doped ZnO (Mo:ZnO). They demonstrated that doping ZnO with molybdenum significantly enhanced its photocatalytic activity, achieving 91% degradation of the dye within just 30 min under ultraviolet irradiation. These findings highlight the potential of modified ZnO-based nanomaterials as effective photocatalysts for the removal of persistent organic pollutants.

Further interest is drawn to the work by More et al. [17], which describes the biomolecule-assisted hydrothermal synthesis of copper aluminum sulfide (CuAlS_2_) nanoparticles. The resulting nanostructures exhibited excellent photocatalytic performance in the degradation of organic dyes, highlighting the potential of sulfide-based systems for incorporation into multifunctional polymeric materials. Such strategies pave the way for the development of environmentally friendly and efficient composite hydrogels capable of acting simultaneously as sorbents and photocatalysts in advanced water purification systems.

However, the application of hydrogels based on unsaturated polyester resins (UPRs) under real-world conditions reveals several significant limitations. First, these materials exhibit limited stability under prolonged exposure to moisture, elevated temperatures, and pH fluctuations, which may result in partial degradation and the loss of sorption capacity. Second, when applied in agricultural environments, such hydrogels are susceptible to biodegradation by the soil microbiota—particularly in the absence of sufficient chemical stabilization of the copolymer structure. This leads to the formation of low-molecular-weight fragments that can migrate into the soil and contribute to the accumulation of secondary degradation products. Third, in wastewater treatment systems, exposure to high flow rates or elevated pressure can compromise the mechanical integrity of hydrogels, especially if they are not reinforced or composited with inorganic fillers.

In addition, challenges also arise in the modification and scalability of such materials.

For instance, achieving uniform distribution of the photocatalyst throughout the hydrogel matrix is technically demanding, particularly at an industrial scale. The presence of inhomogeneous regions can reduce photocatalytic efficiency or lead to localized overheating. Furthermore, the issue of reusability remains unresolved—once saturated with pollutants, many hydrogel types are difficult to regenerate without a significant loss of functionality.

The use of moisture sorbents in agriculture to improve growing conditions for crops has led to the widespread application of hydrogels. However, prolonged use of artificial soils created based on hydrogels may lead to their aging and degradation, which could, in turn, negatively impact crop yields. Currently, we lack sufficient knowledge of substances that could cause the decomposition of polymer gels added to natural soil, as well as the structural components that form the basis of artificial soils under the influence of various environmental factors. A key issue is the identification of these factors, including seasonal and diurnal temperature fluctuations, to prevent the degradation of hydrogels and maintain high crop yields. We have become one of the first research groups worldwide to recognize the importance of addressing these issues.

Different types of polymers exhibit different aging rates, regardless of the level of external factors. Unsaturated polymers (unsaturated polyesters), which contain double bonds in their molecules, have the highest aging rates, while fluorine-containing polymers exhibit the slowest aging. As a result of aging, polymers lose their key properties—elasticity, transparency, color, dielectric properties, and others. The effects of thermal treatment on the supramolecular structure of copolymers based on polypropylene glycol fumarate phthalate and acrylic acid were thoroughly investigated in ref. [18].

Addressing the issues related to the stability and use of hydrogels in soil is impossible without the application of computational methods. Modern computational approaches allow for a detailed study of polymer decomposition kinetics at the molecular level, modeling complex chemical processes that are difficult or impossible to reproduce experimentally. Methods such as molecular modeling [19], molecular dynamics, and quantum chemical calculations [20] enable the investigation of the thermodynamic and kinetic parameters of polymer decomposition, as well as the identification of the impact of various factors, such as temperature, pressure, the presence of catalysts, and other additives. Furthermore, computational methods allow for the prediction of polymer behavior under real-world conditions, which is crucial for the development of new materials with specific properties. This approach can assist in creating more degradation-resistant polymers, a significant task in the search for sustainable and eco-conscious solutions for industry. Moreover, the use of computational models can significantly reduce the time and resources required for experiments, making the research process more efficient and focused. Thus, the integration of computational approaches into the study of polymer decomposition kinetics opens new horizons for scientific research and the development of advanced materials.

The goal of this work is to apply computational methods in a detailed analysis of the decomposition kinetics of copolymers based on polypropylene glycol fumarate phthalate and to develop models that describe these processes. We rely on the results of thermal analysis to establish a connection between the structural features of the polymers and their thermal behavior, which is an important step toward creating more stable and effective materials for industrial applications.

Experimental thermogravimetric data obtained from TGA-DSC-MS and TGA-DSC-IR analyses were employed for comparative evaluation and validation of the numerical results. These measurements provide critical reference points for comparing the predicted decomposition stages with the observed mass loss profiles and thermal events. Although this study is not primarily experimental in nature, the inclusion of benchmark data allows for the evaluation of model accuracy and supports the interpretation of the quantum chemical results. The conditions for thermogravimetric measurements and the calibration procedure were performed in accordance with ASTM E1582-00 [21] and are described in detail in Supplementary Material S1.

## 2. Methods

### 2.1. Approximation Techniques and TGA-Based Analysis

To process the results of thermogravimetric analysis (TGA), the software environments OriginPro 9.0 and Anaconda (Python 3.10 with NumPy, Matplotlib, version 3.10.1, and SciPy libraries) were utilized. The primary objective was to approximate the experimental mass loss curves as a function of temperature. Polynomial regression models implemented in polynomial models from the Polynomial Fit module of OriginPro 9.0 were used to analyze the dependencies, providing both statistical evaluation and visual representation of the results.

Polynomial fitting enabled accurate representation of degradation curve segments and identification of characteristic temperature intervals corresponding to different stages of copolymer decomposition. For each thermogram, an optimal polynomial order was selected by minimizing the sum of squared deviations using the least squares method. The resulting approximation equations were used as input functions for further numerical analysis, including derivative calculation (degradation rate), identification of temperature extrema, and integration into systems of equations that model the kinetic parameters of decomposition.

In addition, Python-based scripts (NumPy/SciPy) were applied to assess the robustness of the fitted models and evaluate their sensitivity to parameter variation, thereby ensuring numerical stability and reducing the influence of random experimental fluctuations. Thus, numerical approximation and TGA data analysis played a pivotal role in constructing reliable computational models and validating the outcomes at subsequent stages of the study.

### 2.2. Density Functional Theory (DFT) Calculations

In this study, density functional theory (DFT) was employed to analyze the molecular structures and evaluate the energetic and spectral properties of the copolymers. DFT is one of the most widely used and reliable quantum chemical approaches for investigating polymer systems. The calculations were performed using the hybrid B3LYP functional in combination with the 6-31G(*d*) basis set, which includes polarization functions on non-hydrogen atoms, as implemented in the Gaussian 16 software package.

The obtained results were used to identify weak chemical bonds that were most susceptible to thermal cleavage. This enabled the interpretation of the stepwise degradation behavior observed in the TGA curves and its correlation with specific molecular fragments.

Thus, DFT modeling using the B3LYP/6-31G(*d*) level of theory provided detailed insights into the electronic, geometric, and spectral properties of the copolymers, offering theoretical support for the experimental observations and numerical kinetic models.

### 2.3. Methods for Solving Systems of Equations

#### 2.3.1. Methods for Calculating Kinetic Parameters

The kinetic mechanism of polymer degradation often follows first-order reaction kinetics with respect to the degree of conversion *α* [22]. The reaction rate constant k is determined by extrapolating the decomposition rate to zero conversion, assuming a linear dependence of *dα*/*dt* on *α*:

(1) $$\frac{d\alpha }{\mathrm{dt}}=k\cdot \left(1-\alpha \right)$$

where *k* is the reaction rate constant.

At high degrees of decomposition, practically every bond breakage leads to the evaporation of chain fragments, which also corresponds to first-order kinetics based on the residual mass of the polymer.

The reaction rate constant *k* is described by the Arrhenius equation:

(2) $$k=A\cdot \exp\left(-\frac{E_{a}}{\mathrm{RT}}\right)$$

where *E_a_*—activation energy, kJ/mol; *A*—pre-exponential factor (frequency factor), min^−1^; *R*—universal gas constant, 8.314 J/(mol·K); and *T*—temperature, *K*.

Then, for the decomposition process:

(3) $$\frac{d\alpha }{\mathrm{dt}}=A\cdot \exp\left(-\frac{E_{a}}{\mathrm{RT}}\right)\left(1-\alpha \right)$$

When the particle mass reaches the value *m_f_*, the reaction stops (*dm*/*dt* = 0). The degree of conversion is calculated using the following formula:

(4) $$\alpha =\frac{m_{0}-m}{m_{0}-m_{f}}$$

where *m*_0_—initial mass of the sample; *m*—current mass; and *m_f_*—final mass.

In non-isothermal analysis with a constant heating rate $H=\frac{\mathrm{dT}}{\mathrm{dt}}$, Equation (3) takes the form:

(5) $$\frac{d\alpha }{\mathrm{dT}}=\frac{A}{H}\exp\left(-\frac{E_{a}}{\mathrm{RT}}\right)\left(1-\alpha \right)$$

Assuming an Arrhenius dependence of the rate constant, integrating Equation (5) gives:

(6) $$g\left(\alpha \right)=\frac{A}{H}\int_{T_{0}}^{T}\exp\left(-\frac{E_{a}}{\mathrm{RT}}\right)\mathrm{dT}$$

With the substitution of variables $x=\frac{E_{a}}{\mathrm{RT}}$ and changing the limits of integration:

(7) $$g\left(\alpha \right)=\frac{\mathrm{AR}}{{\mathrm{HE}}_{a}}\int_{x_{0}}^{x}x^{-2}e^{-x}dx$$

Let us introduce the notation:

(8) $$p\left(x\right)=\int_{x}^{\infty }x^{-2}e^{-x}dx$$

Then, Equation (7) can be written as follows:

(9) $$g\left(\alpha \right)=\frac{\mathrm{AR}}{{\mathrm{HE}}_{a}}p\left(x\right)$$

For approximate calculations, an asymptotic expansion of the integral of the exponential function in Equation (8) is used, which gives:

(10) $$p\left(x\right)\approx \frac{e^{-x}}{x^{2}}$$

Under the condition $\frac{2\mathrm{RT}}{E_{a}}<<1$, the following is true:

(11) $$p\left(\frac{E_{a}}{\mathrm{RT}}\right)\approx \frac{{\mathrm{RT}}^{2}}{E_{a}^{2}}\exp\left(-\frac{E_{a}}{\mathrm{RT}}\right)$$

Then, the equation takes the form:

(12) $$g\left(\alpha \right)=\frac{{\mathrm{ART}}^{2}}{{\mathrm{HE}}_{a}^{2}}\exp\left(-\frac{E_{a}}{\mathrm{RT}}\right)$$

from which we express:

(13) $$\log\;g\left(\alpha \right)=\left(\frac{{\mathrm{ART}}^{2}}{{\mathrm{HE}}_{a}^{2}}\right)-\frac{E_{a}}{\mathrm{RT}}$$

Taking the logarithm of both sides, we obtain a linear dependence:

(14) $$\log\;g\left(\alpha \right)=\left(\frac{{\mathrm{AR}}^{2}}{{\mathrm{HE}}_{a}}\right)-\frac{E_{a}}{\mathrm{RT}}$$

or

(15) $$\log\;g\left(\alpha \right)=C-\frac{E_{a}}{\mathrm{RT}}$$

where

(16) $$g\left(\alpha \right)=\int_{0}^{\alpha }\frac{d\alpha }{{\left(1-\alpha \right)}^{n}}$$

and for the first order (*n* = 1):

(17) $$g\left(\alpha \right)=-\log\left(1-\alpha \right)$$

The system of kinetic equations is then rewritten in matrix form for numerical computation:

*Tx* = *h* (18)

where *T* is the matrix containing temperature data and a unit column, *x* is the vector of unknown parameters *m* and *b*, and *h* is the vector of logarithm values of measured quantities.

Formation of the matrix for the copolymer p-PGFPh:AA (6.77:93.23 mol.%):

$$\left[\begin{array}{cc} T_{1} & 1\\ T_{2} & 1\\ \vdots & \vdots \\ T_{n} & 1 \end{array}\right]\left[\begin{array}{c} m\\ b \end{array}\right]=\left[\begin{array}{c} h_{1}\\ h_{2}\\ \vdots \\ h_{n} \end{array}\right]\Rightarrow \left[\begin{array}{ll} 1.80 & 1\\ 1.79 & 1\\ 1.78 & 1\\ 1.77 & 1\\ 1.76 & 1\\ 1.75 & 1\\ 1.74 & 1\\ 1.74 & 1\\ 1.73 & 1\\ 1.72 & 1\\ 1.71 & 1\\ 1.71 & 1\\ 1.70 & 1\\ 1.69 & 1\\ 1.68 & 1\\ 1.68 & 1\\ 1.67 & 1\\ 1.66 & 1\\ 1.65 & 1\\ 1.65 & 1\\ 1.64 & 1\\ 1.63 & 1\\ 1.63 & 1\\ 1.62 & 1\\ 1.61 & 1\\ 1.61 & 1\\ 1.60 & 1\\ 1.59 & 1\\ 1.59 & 1\\ 1.58 & 1\\ 1.57 & 1 \end{array}\right]\left[\begin{array}{c} m\\ b \end{array}\right]=\left[\begin{array}{c} 0.51\\ 0.58\\ 0.68\\ 0.73\\ 0.76\\ 0.80\\ 0.86\\ 0.94\\ 1.02\\ 1.06\\ 1.12\\ 1.17\\ 1.25\\ 1.40\\ 1.45\\ 1.51\\ 1.62\\ 1.72\\ 1.81\\ 1.90\\ 2.00\\ 2.11\\ 2.20\\ 2.28\\ 2.37\\ 2.46\\ 2.55\\ 2.63\\ 2.68\\ 2.73\\ 2.79 \end{array}\right]\Rightarrow \mathrm{Tx}=h$$

Thus, the obtained expression in matrix form is equivalent to a system of linear equations.

$$\begin{array}{c} m\cdot \frac{1}{T_{1}}+b=h_{1}\\ m\cdot \frac{1}{T_{2}}+b=h_{2}\\ \vdots \\ m\cdot \frac{1}{T_{n}}+b=h_{n} \end{array}$$

#### 2.3.2. Method of Least Squares

Since the experimentally obtained *h* values may not be in the column space of *T*, most experimental systems lack an exact solution. Instead, a least squares vector is computed to minimize the squared distance between the vector and the solution.

In the context of the present study, the method of least squares was employed to determine the coefficients of polynomial models approximating the thermogravimetric decomposition curves. The resulting fitted functions were subsequently used to compute derivatives and reaction rates, which are critical for the evaluation of kinetic parameters. This method was chosen for its simplicity, mathematical rigor, broad applicability, and compatibility with numerical solution techniques such as QR, SVD, and Cholesky decompositions, which ensure computational stability even in the presence of multicollinearity or overdetermined systems.

The least squares method (LSM) involves solving the equation:

(19) $$Q\left(b,m,T_{i}\right)=\frac{1}{n}\sum_{i=1}^{n}{\left(b+{\mathrm{mT}}_{i}-y_{i}\right)}^{2}$$

To do this, we need to:

- Find the partial derivatives of the function *Q*(*b, m* and *T_i_*) to the unknowns *b* and *m*;
- Set the resulting expressions equal to zero;
- Solve the resulting system of two equations with two unknowns.

The solution to this system of equations will provide the desired model parameters.

Let us describe the analytical solution to this system in detail. We will begin by calculating the derivative of the function *Q*(*b, m* and *T_i_*) to the parameter *b*.

We start with the application of the sum rule for differentiation:

$$\frac{\partial Q(b,m,T_{i})}{\partial b}=\frac{\partial \left(\frac{1}{n}\sum_{i=1}^{n}{\left(b+{\mathrm{mT}}_{i}-y_{i}\right)}^{2}\right)}{\partial b}=\frac{1}{n}\sum_{i=1}^{n}\frac{\partial {\left(b+{\mathrm{mT}}_{i}-y_{i}\right)}^{2}}{\partial b}=$$

Next, we apply the chain rule for differentiating a composite function:

$$=\frac{1}{n}\sum_{i=1}^{n}\frac{\partial {\left(b+{\mathrm{mT}}_{i}-y_{i}\right)}^{2}}{\partial \left(b+{\mathrm{mT}}_{i}-y_{i}\right)}\cdot \frac{\partial \left(b+{\mathrm{mT}}_{i}-y_{i}\right)}{\partial b}=$$

Then, we apply the sum rule for differentiating a function presented as a sum:

$$\begin{array}{c} =\frac{1}{n}\sum_{i=1}^{n}2\cdot \left(b+{\mathrm{mT}}_{i}-y_{i}\right)\cdot \left(\frac{\partial b}{\partial b}+\frac{\partial \left({\mathrm{mT}}_{i}\right)}{\partial b}-\frac{\partial y_{i}}{\partial b}\right)=\\ =\frac{2}{n}\sum_{i=1}^{n}\left(b+{\mathrm{mT}}_{i}-y_{i}\right)\cdot \left(1+0-0\right)=\frac{2}{n}\sum_{i=1}^{n}\left(b+{\mathrm{mT}}_{i}-y_{i}\right)= \end{array}$$

Now, we compute the derivative of the function *Q(b, m, and T_i_)* with respect to the parameter *m* and apply the sum rule for differentiation:

$$\frac{\partial Q\left({b,m,T}_{i}\right)}{\partial m}=\frac{\partial \left(\frac{1}{n}\sum_{i=1}^{n}{\left(b+{\mathrm{mT}}_{i}-y_{i}\right)}^{2}\right)}{\partial m}=\frac{1}{n}\sum_{i=1}^{n}\frac{\partial {\left(b+{\mathrm{mT}}_{i}-y_{i}\right)}^{2}}{\partial m}=$$

Next, we apply the chain rule for differentiating a composite function:

$$=\frac{1}{n}\sum_{i=1}^{n}\frac{\partial {\left(b+{\mathrm{mT}}_{0}-y_{i}\right)}^{2}}{\partial \left(b+{\mathrm{mT}}_{0}-y_{i}\right)}\cdot \frac{\partial \left(b+{\mathrm{mT}}_{0}-y_{i}\right)}{\partial m}=$$

We then apply the sum rule for differentiation:

(20) $$\begin{array}{c} =\frac{1}{n}\sum_{i=1}^{n}2\cdot \left(b+{\mathrm{mT}}_{i}-y_{i}\right)\cdot \left(\frac{\partial b}{\partial m}+\frac{\partial \left({\mathrm{mT}}_{i}\right)}{\partial m}-\frac{\partial y_{i}}{\partial m}\right)=\\ =\frac{2}{n}\sum_{i=1}^{n}\left(b+{\mathrm{mT}}_{i}-y_{i}\right)\cdot \left(0+T_{i}-0\right)=\frac{2}{n}\sum_{i=1}^{n}\left(\left(b+w_{1}^{*}T_{i}-y_{i}\right)\cdot T_{i}\right). \end{array}$$

Now, we set the resulting derivatives equal to zero and solve the resulting system of equations:

(21) $$\left\{\begin{array}{l} \frac{2}{n}\sum_{i=1}^{n}\left(b+{\mathrm{mT}}_{i}-y_{i}\right)=0,\\ \frac{2}{n}\sum_{i=1}^{n}\left(\left(b+{\mathrm{mT}}_{i}-y_{i}\right)\cdot T_{i}\right)=0. \end{array}\right.$$

We expand the brackets:

(22) $$\left\{\begin{array}{l} \sum_{i=1}^{n}b+\sum_{i=1}^{n}{\mathrm{mT}}_{i}-\sum_{i=1}^{n}y_{i}=0,\\ \sum_{i=1}^{n}{\mathrm{bT}}_{i}+\sum_{i=1}^{n}{\mathrm{mT}}_{i}^{2}-\sum_{i=1}^{n}y_{i}T_{i}=0. \end{array}\right.$$

We factor out the constant multipliers:

(23) $$\left\{\begin{array}{l} n\cdot b+m\sum_{i=1}^{n}T_{i}-\sum_{i=1}^{n}y_{i}=0,\\ b\sum_{i=1}^{n}T_{i}+m\sum_{i=1}^{n}T_{i}^{2}-\sum_{i=1}^{n}y_{i}T_{i}=0. \end{array}\right.$$

We move the terms with the “*y*” multiplier to the right-hand side of the equations:

(24) $$\left\{\begin{array}{l} n\cdot b+m\sum_{i=1}^{n}T_{i}=\sum_{i=1}^{n}y_{i},\\ b\sum_{i=1}^{n}T_{i}+m\sum_{i=1}^{n}T_{i}^{2}=\sum_{i=1}^{n}y_{i}T_{i}. \end{array}\right.$$

We arrange the terms with the “*x*” multiplier on the left-hand side in descending order of powers:

(25) $$\left\{\begin{array}{l} m\sum_{i=1}^{n}T_{i}^{2}+b\sum_{i=1}^{n}T_{i}=\sum_{i=1}^{n}T_{i}y_{i},\\ b\sum_{i=1}^{n}T_{i}+n\cdot w_{0}^{*}=\sum_{i=1}^{n}y_{i}. \end{array}\right.$$

To solve the resulting system of algebraic equations, we represent it in matrix form:

(26) $$\left(\begin{array}{cc} \sum_{i=1}^{n}T_{i}^{2} & \sum_{i=1}^{n}T_{i}\\ \sum_{i=1}^{n}T_{i} & n \end{array}\right)\left(\begin{array}{c} m\\ b \end{array}\right)=\left(\begin{array}{c} \sum_{i=1}^{n}y_{i}T_{i}\\ \sum_{i=1}^{n}y_{i} \end{array}\right)$$

We express the vector *w*^∗^ with the desired weights by multiplying both sides of the equation by the inverse matrix of y:

(27) $$\left(\begin{array}{c} m\\ b \end{array}\right)={\left(\begin{array}{cc} \sum_{i=1}^{n}T_{i}^{2} & \sum_{i=1}^{n}T_{i}\\ \sum_{i=1}^{n}T_{i} & n \end{array}\right)}^{-1}\left(\begin{array}{c} \sum_{i=1}^{n}y_{i}T_{i}\\ \sum_{i=1}^{n}y_{i} \end{array}\right)$$

The resulting expression (26) is the solution to the system of equations and can now be used as the first algorithm for estimating the model parameters. 

The Python implementation of the least squares method is available in Supplementary Material S2.

#### 2.3.3. Normal Equations

In this study, the normal equations method was used to determine the thermodynamic parameters of the synthesized copolymers. This method minimizes the quadratic approximation error by solving the following equation:

**Theorem** **1.** *The least squares solution to* T(x)=h *is also a solution to* T*Tx=T*h *in the normal equations, where the function* $r(x)={\left\|\mathrm{Tx}-h\right\|}^{2}$ *is minimized [23].*

**Proof.**  The vector h can be decomposed into component vectors where $h_{\|}$ is in the column space of T and $h_{⊥}$ is orthogonal $h_{\|}$ and every column of T, $h=h_{\|}+h_{⊥}$. Therefore, $T*h_{⊥}=0$. □

Suppose *x* is a least squares solution. Then,

$$T*Tx=T*h_{\|}$$

(28) $$\begin{array}{l} =T*(h-h_{⊥})\\ =T*h-T*h_{⊥}\\ =T*h \end{array}$$

So *x* is a solution to the equation T*Tx=T*h. If *T* is full rank, then T*T and the system of normal equations are nonsingular and *x* is a unique least squares solution [24].

In this case, because the rate reactions and temperature are the only two variables measured experimentally, *T* will always have size m≥n, where n is always 2 and *m* is equal to the number of data points collected.

**Theorem** **2.** *If* T *is size* m×n *with* m≥n*, then* T *has full rank if and only if its columns form a linearly independent set.*

**Proof.**  $\left(\Rightarrow \right)$ If *T* has full rank, then by definition its rank is equal to *n*. From basic knowledge we know that *n* equals the rank plus the nullity of *T* where the nullity is the dimension of the null space of *T*. In this case, since the rank is equal to *n*, the nullity of *T* is zero. Therefore *T* is nonsingular, and from the properties of a nonsingular matrix we known the columns of *T* form a linearly independent set. $\left(⇐\right)$ If the columns of *T* are linearly independent, then the nullity of *T* is zero. Then, $r\left(T\right)+0=n$, so the rank of *T* is equal to the number of columns *n*. So by definition *T* is full rank. □

Conclusion: As shown in the construction of the system Tx=h, the first column of *T* holds the experimental temperatures and the second column has ones in every entry. Thus there is no scalar that can take the first column to the second, so the columns of *T* will always form a linearly independent set. Then, by Theorem 2, T will always have full rank. Because *T* will always have full rank, the least squares solution ***x*** will always be unique by Theorem 1. 

To determine the thermodynamic parameters of the synthesized copolymers, the method of normal equations is used:

T*Tx=T*h

$$\begin{array}{cc} {\left[\begin{array}{ll} 1.80 & 1\\ 1.79 & 1\\ 1.78 & 1\\ 1.77 & 1\\ 1.76 & 1\\ 1.75 & 1\\ 1.74 & 1\\ 1.74 & 1\\ 1.73 & 1\\ 1.72 & 1\\ 1.71 & 1\\ 1.71 & 1\\ 1.70 & 1\\ 1.69 & 1\\ 1.68 & 1\\ 1.68 & 1\\ 1.67 & 1\\ 1.66 & 1\\ 1.65 & 1\\ 1.65 & 1\\ 1.64 & 1\\ 1.63 & 1\\ 1.63 & 1\\ 1.62 & 1\\ 1.61 & 1\\ 1.61 & 1\\ 1.60 & 1\\ 1.59 & 1\\ 1.59 & 1\\ 1.58 & 1\\ 1.57 & 1 \end{array}\right]}^{*}\left[\begin{array}{ll} 1.80 & 1\\ 1.79 & 1\\ 1.78 & 1\\ 1.77 & 1\\ 1.76 & 1\\ 1.75 & 1\\ 1.74 & 1\\ 1.74 & 1\\ 1.73 & 1\\ 1.72 & 1\\ 1.71 & 1\\ 1.71 & 1\\ 1.70 & 1\\ 1.69 & 1\\ 1.68 & 1\\ 1.68 & 1\\ 1.67 & 1\\ 1.66 & 1\\ 1.65 & 1\\ 1.65 & 1\\ 1.64 & 1\\ 1.63 & 1\\ 1.63 & 1\\ 1.62 & 1\\ 1.61 & 1\\ 1.61 & 1\\ 1.60 & 1\\ 1.59 & 1\\ 1.59 & 1\\ 1.58 & 1\\ 1.57 & 1 \end{array}\right] & \left[\begin{array}{c} m\\ b \end{array}\right] \end{array}={\left[\begin{array}{ll} 1.80 & 1\\ 1.79 & 1\\ 1.78 & 1\\ 1.77 & 1\\ 1.76 & 1\\ 1.75 & 1\\ 1.74 & 1\\ 1.74 & 1\\ 1.73 & 1\\ 1.72 & 1\\ 1.71 & 1\\ 1.71 & 1\\ 1.70 & 1\\ 1.69 & 1\\ 1.68 & 1\\ 1.68 & 1\\ 1.67 & 1\\ 1.66 & 1\\ 1.65 & 1\\ 1.65 & 1\\ 1.64 & 1\\ 1.63 & 1\\ 1.63 & 1\\ 1.62 & 1\\ 1.61 & 1\\ 1.61 & 1\\ 1.60 & 1\\ 1.59 & 1\\ 1.59 & 1\\ 1.58 & 1\\ 1.57 & 1 \end{array}\right]}^{*}\left[\begin{array}{c} 0.51\\ 0.58\\ 0.68\\ 0.73\\ 0.76\\ 0.80\\ 0.86\\ 0.94\\ 1.02\\ 1.06\\ 1.12\\ 1.17\\ 1.25\\ 1.40\\ 1.45\\ 1.51\\ 1.62\\ 1.72\\ 1.81\\ 1.90\\ 2.00\\ 2.11\\ 2.20\\ 2.28\\ 2.37\\ 2.46\\ 2.55\\ 2.63\\ 2.68\\ 2.73\\ 2.79 \end{array}\right]$$

$$\begin{array}{cc} \left[\begin{array}{cc} 91.17 & 53.97\\ 53.97 & 32.00 \end{array}\right] & \left[\begin{array}{c} m\\ b \end{array}\right] \end{array}=\left[\begin{array}{c} 87.27\\ 52.69 \end{array}\right]$$

*m* = −10.73 ⇒ *E_a_ = 2.3mR* = 205.22 kJ/mol

*b* = 19.75 ⇒ *A* = *e^b^* = 3.77 × 10^8^

#### 2.3.4. Cholesky Factorization

A method analogous to the use of normal equations in least squares problems is Cholesky decomposition.

When deriving normal equations, matrix A, resulting from the product of the coefficient matrix and its transpose (i.e., Tᵀ·T), is symmetric.

Moreover, in Arrhenius-based calculations, the temperature is expressed in kelvin (e.g., 0 °C = 273 K), which ensures that the elements of matrix A remain positive.

This method is commonly used to solve linear systems arising from error minimization in regression problems.

In the present study, Cholesky decomposition was employed to solve algebraic systems derived from the approximation of experimental thermogravimetric data.

It was selected for its computational efficiency, low memory requirements, and high numerical stability—particularly advantageous when dealing with symmetric matrices, as typically encountered in modeling thermal decomposition processes.

**Definition** **1** ([25])**.** *If* $\langle x,Ax\rangle \geq 0$ *for all x, then A is a symmetric positive definite matrix where* x≠0.

Therefore, by definition, T*T is a symmetric positive definite matrix.

**Theorem** **3.** *If* T*T *is a symmetric positive definite matrix, then there exists a unique upper triangular matrix G with positive diagonal entries, such that* T*T=G*G *[26].*

**Proof.**  Since the matrix is symmetric and positive definite, it can be decomposed into a block diagonal matrix, where the first block is a matrix. By performing row operations and subsequent column operations, it can be transformed into an identity matrix. The cumulative effect of the row operations accumulates in the lower triangular matrix, while the cumulative effect of the column operations remains in the upper triangular matrix. □

In this case, the original matrix A is transformed in block form:

$$T*T=A=\left[\begin{array}{cc} a & y*\\ y & B \end{array}\right]$$

For this structure, decomposition into a product of matrices of the form is used:

(29) $$\begin{array}{c} T*T=A=\left[\begin{array}{cc} \sqrt{a} & 0^{*}\\ \frac{1}{\sqrt{a}}y & I \end{array}\right]\left[\begin{array}{cc} 1 & 0^{*}\\ 0 & B-\frac{1}{a}yy^{*} \end{array}\right]\left[\begin{array}{cc} \sqrt{a} & \frac{1}{\sqrt{a}}y^{*}\\ 0 & I \end{array}\right]\\ =G*A_{1}G_{1} \end{array}$$

Since T*T is a Hermitian matrix, it will always have real diagonal elements. Therefore, a will always be positive. The repeated decomposition of the matrix $B-\frac{1}{a}\mathrm{yy}*$ ultimately yields the identity matrix. The element in the top right corner of this matrix should always be positive because T*T is symmetric and positive definite:

$$a=\langle e_{2},A_{1}G_{1}^{-1}e_{2}\rangle \geq 0\;x=G_{1}^{-1}e_{2}$$

After interactions n:

$$A=G_{n}^{*}\ldots G_{2}^{*}G_{1}^{*}IG_{1}G_{2}\ldots G_{n}=G*G$$

Then [26,27],

(30) T*T=G*G→G*Gx=T*h

Using Cholesky decomposition to determine the thermodynamic parameters of the synthesized copolymer p-PGFPh:AA (6.77:93.23 mol.%), we can observe that:

$$T*T={\left[\begin{array}{ll} 1.80 & 1\\ 1.79 & 1\\ 1.78 & 1\\ 1.77 & 1\\ 1.76 & 1\\ 1.75 & 1\\ 1.74 & 1\\ 1.74 & 1\\ 1.73 & 1\\ 1.72 & 1\\ 1.71 & 1\\ 1.71 & 1\\ 1.70 & 1\\ 1.69 & 1\\ 1.68 & 1\\ 1.68 & 1\\ 1.67 & 1\\ 1.66 & 1\\ 1.65 & 1\\ 1.65 & 1\\ 1.64 & 1\\ 1.63 & 1\\ 1.63 & 1\\ 1.62 & 1\\ 1.61 & 1\\ 1.61 & 1\\ 1.60 & 1\\ 1.59 & 1\\ 1.59 & 1\\ 1.58 & 1\\ 1.57 & 1 \end{array}\right]}^{*}\left[\begin{array}{ll} 1.80 & 1\\ 1.79 & 1\\ 1.78 & 1\\ 1.77 & 1\\ 1.76 & 1\\ 1.75 & 1\\ 1.74 & 1\\ 1.74 & 1\\ 1.73 & 1\\ 1.72 & 1\\ 1.71 & 1\\ 1.71 & 1\\ 1.70 & 1\\ 1.69 & 1\\ 1.68 & 1\\ 1.68 & 1\\ 1.67 & 1\\ 1.66 & 1\\ 1.65 & 1\\ 1.65 & 1\\ 1.64 & 1\\ 1.63 & 1\\ 1.63 & 1\\ 1.62 & 1\\ 1.61 & 1\\ 1.61 & 1\\ 1.60 & 1\\ 1.59 & 1\\ 1.59 & 1\\ 1.58 & 1\\ 1.57 & 1 \end{array}\right]=\left[\begin{array}{cc} 91.17 & 53.97\\ 53.97 & 32.00 \end{array}\right]$$

where T*T=$T^{T}T$

$$\begin{array}{l} T*T=\left[\begin{array}{cc} 91.17 & 53.97\\ 53.97 & 32.00 \end{array}\right]\\ =\begin{array}{ccc} \left[\begin{array}{cc} \sqrt{91.17} & 0\\ \frac{53.97}{\sqrt{91.17}} & 1 \end{array}\right] & \left[\begin{array}{cc} 1 & 0\\ 0 & 32-\frac{53.97^{2}}{91.17} \end{array}\right] & \left[\begin{array}{cc} \sqrt{91.17} & \frac{53.97}{\sqrt{91.17}}\\ 0 & 1 \end{array}\right] \end{array}\\ =\begin{array}{ccc} \left[\begin{array}{cc} \sqrt{91.17} & 0\\ \frac{53.97}{\sqrt{91.17}} & 32.00-\frac{53.97^{2}}{91.17} \end{array}\right] & \left[\begin{array}{cc} 1 & 0\\ 0 & 1 \end{array}\right] & \left[\begin{array}{cc} \sqrt{91.17} & \frac{53.97}{\sqrt{91.17}}\\ 0 & 32.00-\frac{53.97^{2}}{91.17} \end{array}\right] \end{array}\\ \;\;\;\;=\begin{array}{cc} \left[\begin{array}{cc} 9.54 & 0\\ 5.65 & 0.22 \end{array}\right] & \left[\begin{array}{cc} 9.54 & 5.65\\ 0 & 0.22 \end{array}\right] \end{array}=G*G \end{array}$$

We solve the system G*w=T*h for w by increasing the matrix size G* with the vector h and reducing the number of rows. Then, we can find the solution to the problem using the least squares method by solving the system Gx=w for x by increasing the matrix size G with the vector w and reducing the number of rows. This will lead to: 

*m* = −10.83 ⇒ *E_a_ = 2.3mR* = 207.21 kJ/mol

*b* = 19.75 ⇒ *A* = *e^b^* = 4.47 × 10^8^

#### 2.3.5. QR Decomposition

The QR decomposition method based on the Gram–Schmidt orthogonalization procedure provides an efficient approach to solving least squares problems for arbitrary coefficient matrices, making it particularly versatile for handling systems of linear equations.

In the present study, QR decomposition was applied to solve algebraic systems arising from multivariate approximation of experimental thermogravimetric curves, as well as in the computation of mathematical model parameters.

The method was chosen for its high numerical stability, especially in cases involving overdetermined systems or correlated variables.

This ensured accurate curve fitting and computational robustness, even in the presence of experimental noise and deviations.

**Theorem** **4.** (Gram–Schmidt Procedure) ([28])**.** *Let* $S=\left\{\nu_{1},\nu_{2},\nu_{3}\ldots \nu_{p}\right\}$ *be a set of linearly independent vectors. Define the vectors* $u_{i}$, 1≤i≤p *as follows:*

(31) $$u_{i}=\nu_{i}-\frac{u_{1}^{*}\nu_{i}}{u_{1}^{*}u_{1}}u_{1}-\frac{u_{2}^{*}\nu_{i}}{u_{2}^{*}u_{2}}u_{2}-\frac{u_{3}^{*}\nu_{i}}{u_{3}^{*}u_{3}}u_{3}-\ldots -\frac{u_{i-1}^{*}\nu_{i}}{u_{i-1}^{*}u_{i-1}}u_{i-1}$$

*Then,* $T=\left\{u_{1},u_{2},u_{3}\ldots u_{p}\right\}$ *is an orthogonal set of non-zero vectors, and* $\langle T\rangle =\langle S\rangle$.

**Theorem** **5** ([25,26,27,28])**.** Let T be m × n matrix of n rank. Then, there exists m × n* Q matrix, the columns of which form an orthogonal set and an upper triangular R matrix of size n with positive diagonal elements, such that T=QR.

**Proof.**  The Gram–Schmidt procedure takes any matrix and produces a set of orthogonal vectors as the columns of the matrix. Each vector of the resulting set is a linear combination of the columns of the original matrix. The coefficients of each linear combination are stored in the upper triangular *R* matrix. Thus, when each orthogonal vector is calculated as the inverse of its norm to become orthonormal, *R* matrix preserves these operations:

$$\begin{array}{l} T=\left[\left.t_{1}\right|t_{2}\right]\\ =\left[\left.u_{1}\right|u_{2}\right]\left[\begin{array}{cc} 1 & \frac{-t_{1}^{*}t_{2}}{t_{1}^{*}t_{1}}\\ 0 & 1 \end{array}\right]\\ =\left[\left.q_{1}\right|q_{2}\right]\left[\begin{array}{cc} \frac{1}{\left\|u_{1}\right\|} & \frac{-t_{1}^{*}t_{2}}{t_{1}^{*}t_{2}}\\ 0 & \frac{1}{\left\|u_{2}\right\|} \end{array}\right] \end{array}$$

□

#### 2.3.6. Modified Gram–Schmidt

The Gram–Schmidt procedure is a vector orthogonalization method widely used in linear algebra. Its purpose is to transform a set of linearly independent vectors into an orthonormal basis. However, the classical Gram–Schmidt algorithm can suffer from numerical instability when dealing with nearly linearly dependent vectors due to the accumulation of rounding errors. The modified version of the algorithm (Modified Gram–Schmidt, MGS) implements the same concept but performs orthogonalization element-wise, subtracting projections immediately, which significantly improves numerical stability.

In the present study, the modified Gram–Schmidt method was employed to construct orthonormal bases in QR decomposition procedures and for preprocessing matrices derived from the approximation of TGA data.

This method was chosen for its enhanced resistance to rounding errors, especially under conditions of strong correlation between variables, which is critical when analyzing experimental data containing noise.

*T* = *QR* (32)

where Q has orthonormal columns and R is the square of the upper triangular matrix of full rank. From the normal equations, we see:

T*Tx=T*h

Q*Q*Rx=R*Q*h

(33) Rx=Q*h-

$$\left[\begin{array}{ll} 1.80 & 1\\ 1.79 & 1\\ 1.78 & 1\\ 1.77 & 1\\ 1.76 & 1\\ 1.75 & 1\\ 1.74 & 1\\ 1.74 & 1\\ 1.73 & 1\\ 1.72 & 1\\ 1.71 & 1\\ 1.71 & 1\\ 1.70 & 1\\ 1.69 & 1\\ 1.68 & 1\\ 1.68 & 1\\ 1.67 & 1\\ 1.66 & 1\\ 1.65 & 1\\ 1.65 & 1\\ 1.64 & 1\\ 1.63 & 1\\ 1.63 & 1\\ 1.62 & 1\\ 1.61 & 1\\ 1.61 & 1\\ 1.60 & 1\\ 1.59 & 1\\ 1.59 & 1\\ 1.58 & 1\\ 1.57 & 1 \end{array}\right]\cdot \left[\begin{array}{cc} 1 & \frac{-t_{1}^{*}t_{2}}{t_{1}^{*}t_{2}}\\ 0 & 1 \end{array}\right]=\left[\begin{array}{cc} 1.80 & 0.18\\ 1.79 & 0.18\\ 1.78 & 0.18\\ 1.77 & 0.18\\ 1.76 & 0.18\\ 1.75 & 0.18\\ 1.74 & 0.18\\ 1.74 & 0.18\\ 1.73 & 0.18\\ 1.72 & 0.18\\ 1.71 & 0.18\\ 1.71 & 0.18\\ 1.70 & 0.17\\ 1.69 & 0.17\\ 1.68 & 0.17\\ 1.68 & 0.17\\ 1.67 & 0.17\\ 1.66 & 0.17\\ 1.65 & 0.17\\ 1.65 & 0.17\\ 1.64 & 0.17\\ 1.63 & 0.17\\ 1.63 & 0.17\\ 1.62 & 0.17\\ 1.61 & 0.17\\ 1.61 & 0.16\\ 1.60 & 0.16\\ 1.59 & 0.16\\ 1.59 & 0.16\\ 1.58 & 0.16 \end{array}\right]\begin{array}{c} {\mathrm{TR}}_{2}=U \end{array}$$

The system Rx=Q*h is nonsingular when *T* has full rank [29].

This method always provides a unique solution to the least squares problem because, according to Theorem 2, *T* always has full rank. Using QR decomposition to determine the thermodynamic parameters of the synthesized copolymer p-PGFPh:AA (6.77:93.23 mol.%):

$$\left[\begin{array}{ll} 1.80 & 1\\ 1.79 & 1\\ 1.78 & 1\\ 1.77 & 1\\ 1.76 & 1\\ 1.75 & 1\\ 1.74 & 1\\ 1.74 & 1\\ 1.73 & 1\\ 1.72 & 1\\ 1.71 & 1\\ 1.71 & 1\\ 1.70 & 1\\ 1.69 & 1\\ 1.68 & 1\\ 1.68 & 1\\ 1.67 & 1\\ 1.66 & 1\\ 1.65 & 1\\ 1.65 & 1\\ 1.64 & 1\\ 1.63 & 1\\ 1.63 & 1\\ 1.62 & 1\\ 1.61 & 1\\ 1.61 & 1\\ 1.60 & 1\\ 1.59 & 1\\ 1.59 & 1\\ 1.58 & 1\\ 1.57 & 1 \end{array}\right]\cdot \left[\begin{array}{cc} \frac{1}{\left\|u_{1}\right\|} & \frac{-t_{1}^{*}t_{2}}{t_{1}^{*}t_{2}}\\ 0 & \frac{1}{\left\|u_{1}\right\|} \end{array}\right]=\left[\begin{array}{cc} -0.30 & 0.93\\ -0.28 & -0.12\\ -0.25 & -0.12\\ -0.23 & -0.11\\ -0.21 & -0.10\\ -0.19 & -0.10\\ -0.17 & -0.09\\ -0.15 & -0.08\\ -0.13 & -0.08\\ -0.11 & -0.07\\ -0.09 & -0.06\\ -0.07 & -0.06\\ -0.05 & -0.05\\ -0.03 & -0.04\\ -0.01 & -0.04\\ 4.89\cdot 10^{-3} & -0.03\\ 0.02 & -0.02\\ 0.04 & -0.02\\ 0.06 & -0.01\\ 0.08 & -9.07\cdot 10^{-3}\\ 0.09 & -2.92\cdot 10^{-3}\\ 0.11 & 3.17\cdot 10^{-3}\\ 0.13 & 9.20\cdot 10^{-3}\\ 0.15 & 0.01\\ 0.17 & 0.02\\ 0.18 & 0.02\\ 0.20 & 0.03\\ 0.22 & 0.03\\ 0.24 & 0.04\\ 0.25 & 0.05\\ 0.27 & 0.05 \end{array}\right]{\mathrm{TR}}_{1}=Q$$

$$T={\mathrm{QR}}_{1}^{-1}=\left[\begin{array}{cc} -0.30 & 0.93\\ -0.28 & -0.12\\ -0.25 & -0.12\\ -0.23 & -0.11\\ -0.21 & -0.10\\ -0.19 & -0.10\\ -0.17 & -0.09\\ -0.15 & -0.08\\ -0.13 & -0.08\\ -0.11 & -0.07\\ -0.09 & -0.06\\ -0.07 & -0.06\\ -0.05 & -0.05\\ -0.03 & -0.04\\ -0.01 & -0.04\\ 4.89\cdot 10^{-3} & -0.03\\ 0.02 & -0.02\\ 0.04 & -0.02\\ 0.06 & -0.01\\ 0.08 & -9.07\cdot 10^{-3}\\ 0.09 & -2.92\cdot 10^{-3}\\ 0.11 & 3.17\cdot 10^{-3}\\ 0.13 & 9.20\cdot 10^{-3}\\ 0.15 & 0.01\\ 0.17 & 0.02\\ 0.18 & 0.02\\ 0.20 & 0.03\\ 0.22 & 0.03\\ 0.24 & 0.04\\ 0.25 & 0.05\\ 0.27 & 0.05 \end{array}\right]\cdot \left[\begin{array}{cc} 9.54 & 5.65\\ 0 & 0.22 \end{array}\right]=\mathrm{QR}$$

We calculate the vector product of matrix Q*h=b and then solve the system *RX = b* for *x* by expanding matrix *R* with the vector *b* and reducing the number of rows, performing the following operations:

*m* = −10.78 ⇒ *E_a_ = 2.3mR* = 206.23 kJ/mol

*b* = 19.84 ⇒ *A* = *e^b^* = 4.12 × 10^8^

The QR decomposition using the Gram–Schmidt orthogonalization procedure works successfully with any matrix as input data. Thus, the successful calculation of the solution does not depend on the elements of the matrix. Consequently, it is robust in the presence of rounding errors and is a valuable computational tool for experimental results in general.

#### 2.3.7. Singular Value Decomposition (SVD)

Singular value decomposition (SVD) is a powerful method used in data structure analysis, dimensionality reduction, noise filtering, and solving ill-posed or poorly conditioned systems of linear equations. Unlike QR or LU decompositions, SVD can be applied to any matrix regardless of its shape, rank, or properties. In the present study, SVD was employed to analyze matrices arising from the approximation of thermogravimetric data and the calculation of model parameters under conditions of multicollinearity and degeneracy. The method was chosen for its high numerical stability and its ability to detect linearly dependent or weakly significant variables. Moreover, SVD enables regularization through truncation of small singular values, thereby stabilizing the solutions and minimizing the influence of experimental noise.

**Theorem** **6.** *If T is a real matrix of m × n size, then there exist orthogonal matrices:*

(34) $$U=\left[\left.u_{1}\right|\ldots \left|u_{m}\right.\right]\mathrm{and}\;V=\left[\left.\nu_{1}\right|\ldots \left|\nu_{n}\right.\right]$$

*where* T=USV* S *is a diagonal matrix with diagonal elements* $\sqrt{\delta_{1}},\ldots ,\sqrt{\delta_{n}}$, *where* $\delta_{1},\ldots \delta_{n}$ *are the singular values of matrix* T*T, *and* U *and* V *are orthogonal matrices of dimensions m and n, respectively* [30].

Using singular value decomposition (SVD) to determine the thermodynamic parameters of the polymers, we observe the eigenvalues of T*T, $\delta_{1}$ and $\delta_{2}$. Thus, the singular values of T are $s_{1}=\sqrt{\delta_{1}}=11.09$ and $s_{2}=\sqrt{\delta_{2}}=0.19$. Arranging the singular values as diagonal elements of the matrix gives:

(35) $$S=\left[\begin{array}{c} \left.s_{1}e_{1}\right|s_{2}e_{2} \end{array}\right]=\left[\begin{array}{cc} 11.09 & 0\\ 0 & 0.19\\ 0 & 0\\ 0 & 0\\ 0 & 0\\ \vdots & \vdots \\ 0 & 0 \end{array}\right]$$

The eigenvectors $\delta_{1}$ and $\delta_{2}$ for matrix T*T, $x_{1}$ and $x_{2}$, expressed as the columns of the matrix are:

(36) $$V*=\left[\left.x_{1}\right|x_{2}\right]*=\left[\begin{array}{cc} -9.54 & -5.65\\ -0.10 & 0.16 \end{array}\right]$$

The first two columns of the unitary matrix are $y_{1}=\frac{1}{\sqrt{\delta_{1}}}{Tx}_{1}$ and $y_{2}=\frac{1}{\sqrt{\delta_{2}}}Tx_{2}$, and then the eigenvectors of T*T corresponding to the zero eigenvalues $y_{3}$, $y_{4}$, $y_{5}$, and $y_{6}$ are the remaining columns. Thus, we have:

(37) $$U=\left[\begin{array}{cccccc} \left.y_{1}\right| & \left.y_{2}\right| & \left.y_{3}\right| & \left.y_{4}\right| & \ldots & y_{n} \end{array}\right]$$

$$U=\left[\begin{array}{cc} -2.04 & -0.05\\ -2.04 & -0.04\\ -2.03 & -0.04\\ -2.02 & -0.03\\ -2.02 & -0.03\\ -2.01 & -0.03\\ -2.00 & -0.02\\ -2.00 & -0.02\\ -1.99 & -0.01\\ -1.98 & -0.01\\ -1.98 & -0.01\\ -1.97 & -7.03\cdot 10^{-3}\\ -1.96 & -3.19\cdot 10^{-3}\\ -1.96 & -5.98\cdot 10^{-4}\\ -1.95 & 4.35\cdot 10^{-3}\\ -1.94 & 8.08\cdot 10^{-3}\\ -1.94 & 0.01\\ -1.93 & 0.01\\ -1.93 & 0.01\\ -1.92 & 0.02\\ -1.91 & 0.02\\ -1.91 & 0.03\\ -1.90 & 0.03\\ -1.90 & 0.03\\ -1.89 & 0.04\\ -1.88 & 0.04\\ -1.88 & 0.04\\ -1.87 & 0.05\\ -1.87 & 0.05\\ -1.86 & 0.05\\ -9.54 & 0.10 \end{array}\right]$$

We solve the system SV*x=U*h for x by augmenting matrix SV* with the vector U*h and reducing the number of rows. However, vector U*h is not in the column space of SV*, and thus the system is inconsistent, representing the 32×3 matrix with ones on the diagonal and values very close to zero elsewhere. To find the solution to the least squares problem, we describe the system in terms of the normal equations. Let C=SV* and b=U*h be given, then we solve the system C*Cx=C*b for the least squares solution of x. This gives:





*m* = −10.78 ⇒ *E_a_ = 2.3mR* = 206.23 kJ/mol

*b* = 19.84 ⇒ *A* = *e^b^* = 4.13 × 10^8^

## 3. Results

It has been previously shown that the thermal decomposition of polymers depends on their chemical composition, degree of crosslinking, and the nature of the monomeric units [1,2,3]. For example, copolymers of unsaturated polyester resins are characterized by high decomposition temperatures (up to 250–300 °C), which is due to their spatially crosslinked structure [4]. At the same time, the mechanism of thermal degradation of such materials remains insufficiently studied, especially in the context of mathematical modeling and numerical analysis. 

**Figure 1:**
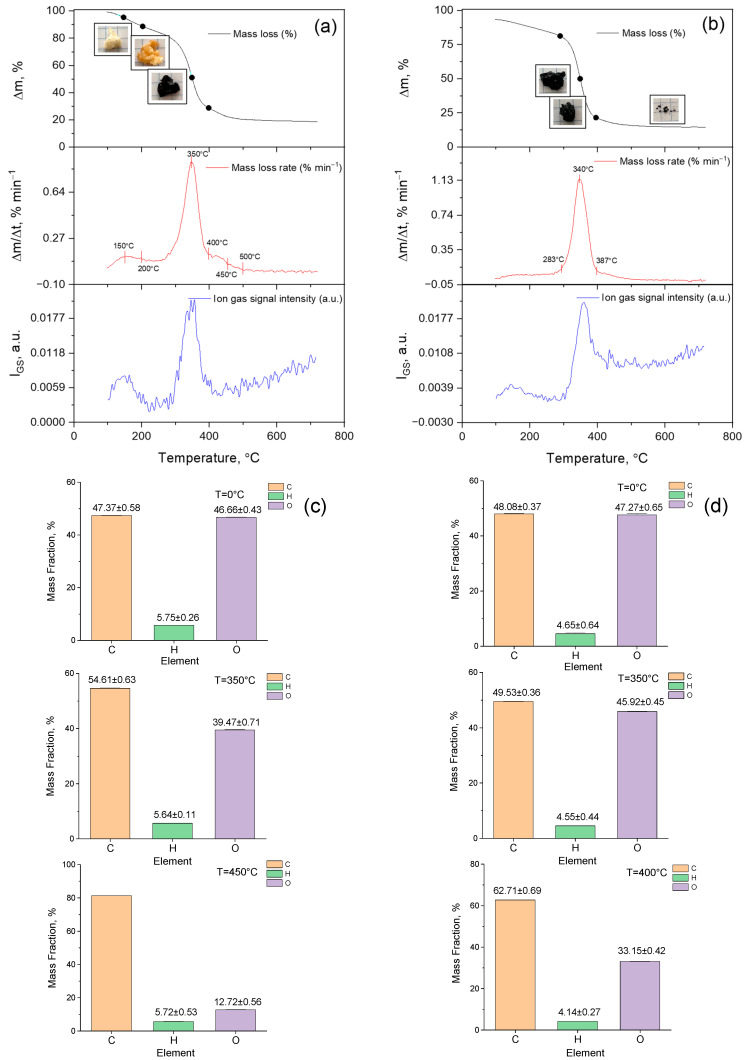
Thermogravimetric analysis (TGA) curves, derivatives of thermogravimetry (DTG) (**a**,**b**), and elemental composition (**c**,**d**) of the p-PGFPh:AA copolymers in a nitrogen atmosphere: (**a**,**c**) composition 6.77:93.23, (**b**,**d**) composition 86.67:13.33.

In this study, the main kinetic parameters of the decomposition of the copolymers p-PGFPh:AA (6.77:93.23) and p-PGFPh:AA (86.67:13.33) in various environments were determined. Mathematical methods for solving linear regression problems were used to process the experimental data, including normal equations, Cholesky decomposition, singular value decomposition (SVD), and QR decomposition. This allowed for the determination of the activation energy (*Eₐ*) and the pre-exponential factor (*A*), as well as confirming the adequacy of the proposed model and its suitability for analyzing the thermal decomposition of copolymers. Thermograms and histograms demonstrating the decomposition rate and elemental composition are presented in Figure 1.

**Figure 2:**
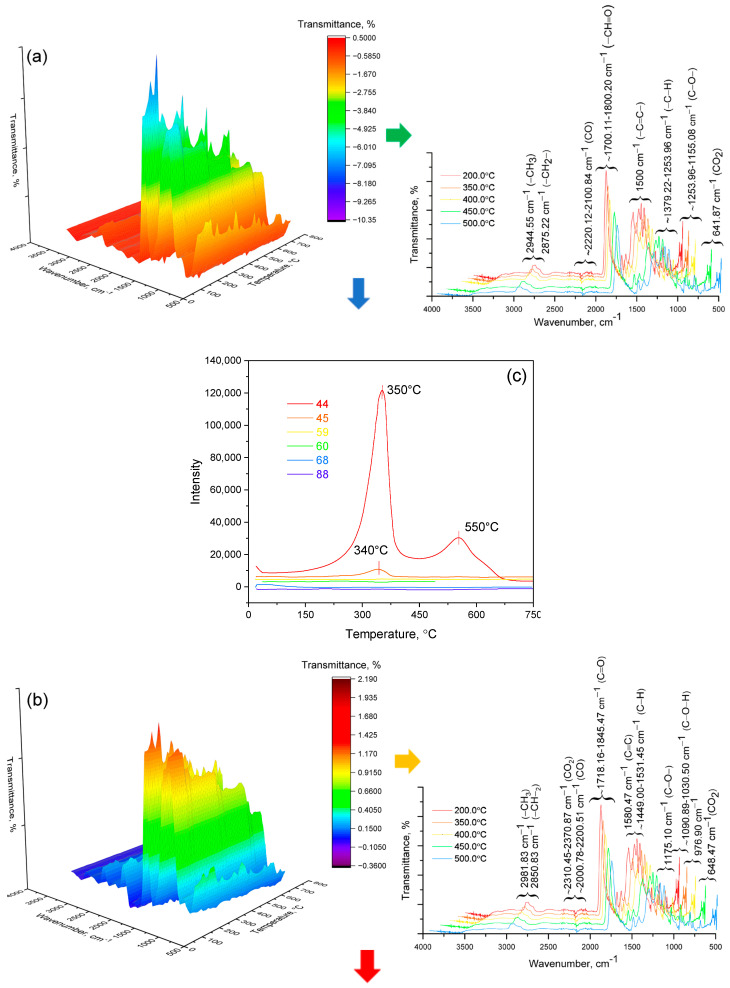

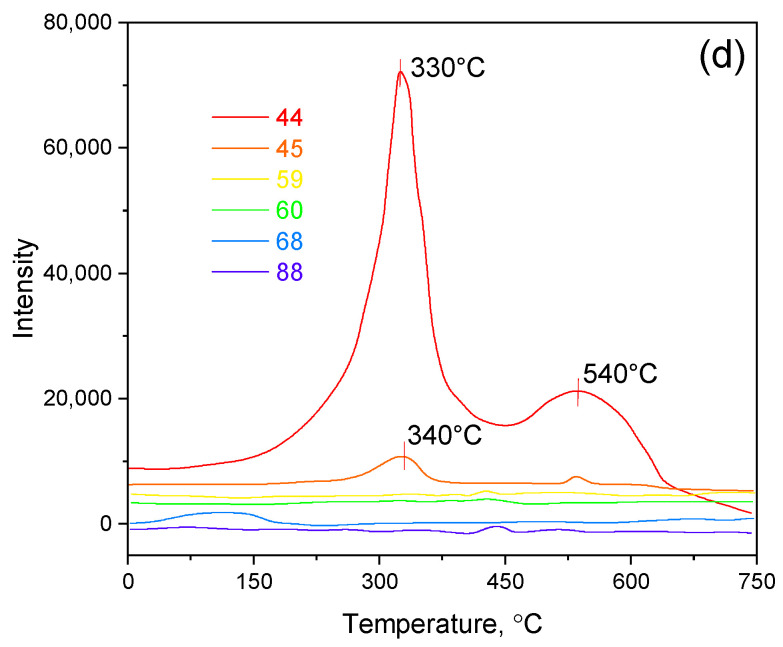
IR (**a**,**b**) and mass spectra (**c**,**d**) of the decomposition products of p-PGFPh:AA at various monomer ratios (M1:M2, mol.%): (**a**,**c**) 6.77:93.23 and (**b**,**d**) 86.67:13.33 recorded at temperatures from 0 to 800 °C. Note: The dominant signal at *m*/*z* = 44 corresponds to CO_2_ evolution, associated with oxidative decarboxylation of carboxylic acid groups. Additional peaks at *m*/*z* = 59 and 60 indicate the release of acetone and acetic acid, while *m*/*z* = 88 reflects heavier oxygenated pyrolysis products from polyester chain breakdown.

As shown in Figure 1a, the decomposition of the copolymer p-PGFPh:AA (6.77:93.23) in a nitrogen atmosphere begins at approximately 100 °C, accompanied by a slight mass loss (∆m = 4.35%) up to about 150 °C, which is due to the evaporation of volatile compounds. The main stage of copolymer decomposition occurs in the range of 250–450 °C, after which the mass stabilizes. At a temperature of 200 °C, the mass loss is 10.07%, at 350 °C it is 48.15%, and at 400 °C it reaches 70.51%, indicating intense decomposition of the polymer matrix. Elemental analysis shows that with increasing temperature, the contents of oxygen and hydrogen decrease, while the proportion of carbon increases, indicating thermo-induced dehydration processes and enrichment of the carbon-containing residue. As seen from the histogram, during the decomposition of the copolymer p-PGFPh:AA (6.77:93.23), oxygen and hydrogen are removed in the form of volatile compounds, with oxygen being released as CO, CO_2_, and H_2_O, while hydrogen is released as H_2_, CH_4_, and other hydrocarbons, as confirmed by the mass spectrometry and IR analysis data (Figure 2).

As a continuation of the study, thermogravimetric investigations of the p-PGFPh:AA copolymers were performed in an air atmosphere. According to the TG curve, mass loss of the sample in air occurs within the temperature range of approximately 130–350 °C. Based on the dTG data, it was established that the main stage of thermal degradation for the copolymer with a composition of 6.77:93.23 mol% proceeds within the range of ~220–350 °C, with a total mass loss of approximately 72.80% of the initial sample mass. Additionally, thermogravimetric analysis was conducted for the copolymer with a composition of 86.67:13.33 mol%. In an air atmosphere, this copolymer undergoes more intense thermal degradation, reaching the maximum decomposition rate at ~287 °C, whereas in an inert environment, the peak shifts to ~340 °C. The thermal curves and corresponding data are provided in Supplementary Material S3.

Figure 1b shows the thermograms of the copolymer p-PGFPh:AA (86.67:13.33), which contains ether (-C-O-C-) linkages connecting fragments of the polymer chain. At 250–340 °C, the breakdown of ether bonds (-C-O-C-) begins, accompanied by the release of volatile products such as formaldehyde (HCHO) and carbonyl compounds (C=O). The rupture of ether linkages leads to the destabilization of the polymer network, initiating the main process of thermal degradation. The formation of low-molecular-weight volatile compounds was confirmed by IR spectroscopy (characteristic C=O and C-O bands) and mass spectrometry (Figure 2).

At 340–450 °C, the degradation of aromatic rings (benzene structures) in the polyester matrix occurs, accompanied by the release of gaseous products such as carbon monoxide (CO), carbon dioxide (CO_2_), toluene (C_6_H_5_CH_3_), and fragments of oligomers with conjugated bonds.

Analysis of the thermogravimetric data revealed fundamental differences in the thermal degradation mechanisms of the p-PGFPh:AA copolymers in air and inert atmospheres. In an air atmosphere, degradation begins at a lower temperature (~287 °C) and is accompanied by the intensive release of oxidation products (CO, CO_2_, and H_2_O), indicating the occurrence of oxidative reactions. By contrast, in a nitrogen atmosphere, decomposition starts at a higher temperature (~340 °C) and proceeds predominantly via a pyrolytic pathway, resulting in the formation of carbonaceous residues. Thus, the presence of oxygen in the reaction environment significantly accelerates the degradation process and alters the decomposition pathway of the polymer matrix, activating radical oxidation and side-chain scission reactions.

### 3.1. Methods of Mathematical Processing of Experimental Data

To determine the kinetic parameters and verify the accuracy of the obtained data, a comparison between the experimental thermogravimetric analysis (TGA) curves and the calculated data was performed. In this context, a framework for modeling the system was developed to enhance the ability to control the structure and molecular properties of the copolymers based on polypropylene glycol fumarate phthalate (p-PGFPh) with acrylic acid. This modeling enables the investigation of the influence of various parameters, such as temperature, thermal treatment time, and the ratio of components, on the thermal degradation process and the formation of the final properties of the material. Moreover, mathematical methods, including numerical calculations and simulations, can help to predict the behavior of the copolymers under different conditions, significantly simplifying the process of optimizing its characteristics for further industrial applications.

**Figure 3:**
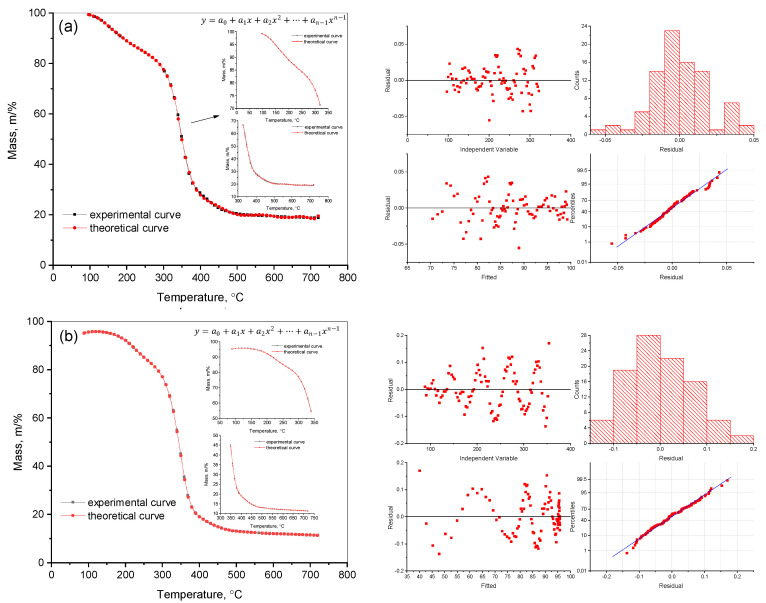
Description of thermograms using mathematical models for the p-PGFPh:AA copolymers with a constant heating rate of 10 °C min^−1^ during two heating cycles: in nitrogen (**a**) and air (**b**).

Copolymers based on polypropylene glycol fumarate phthalate have the potential to exhibit significantly improved thermal and mechanical properties [31]. In light of this, we decided to conduct further studies for a more detailed analysis of these properties. To describe the thermogram [32], we used mathematical models, which allowed for an accurate approximation of the experimental data and the identification of the main patterns in the behaviors of the p-PGFPh copolymers. The mass–temperature curves were approximated using a ninth-degree polynomial model implemented in the Polynomial Fit module of the OriginPro 9.0 software package. This choice was based on the model’s ability to provide the best fit to the experimental thermogravimetric data while maintaining robustness against overfitting. Statistical evaluation of the model via analysis of variance (ANOVA) confirmed its high significance, with an F-statistic of 71,581.57 and a *p*-value < 0.0001. The reduced chi-square value was 4.02 × 10^−4^, indicating minimal residual deviations. The coefficient of determination R^2^ and the adjusted R^2^ both reached 0.99999, demonstrating almost perfect agreement between the model and the experimental curves (Figure 3). This approach not only improved the accuracy of data processing but also enabled a deeper interpretation of the kinetic characteristics.

**Figure 4:**
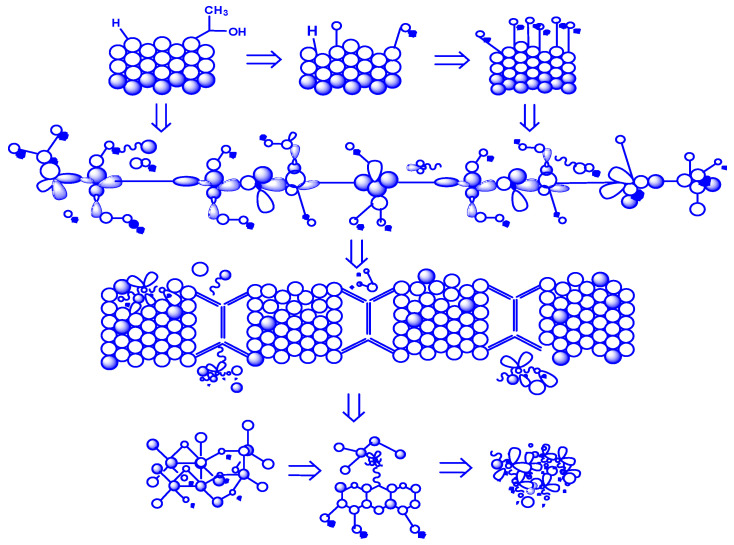
Mechanism of degradation of the p-PGFPh:AA copolymers under the influence of temperature.

The mechanism of thermal degradation of the copolymer of polypropylene glycol fumarate phthalate with acrylic acid, with a composition of 6.77:93.23 mol.%, is presented in the form of a sequence of stages reflecting the main degradation pathways (Figure 4). The following key stages of the thermal decomposition of the copolymer are shown in Figure 4:

(1) Initiation—The initial stage, involving the rupture of weak bonds in the polymer chain, which triggers the process of technological degradation.
(2) Propagation—The process in which active radicals are formed, stimulating further polymer breakdown. As a result of structural changes in the chain, degradation products begin to form.
(3) Elimination—The stage in which volatile compounds, such as carbon dioxide (CO_2_), carbon monoxide (CO), and other small molecules, are formed.
(4) Formation of Intermediate Products—During thermal degradation, cyclic and aromatic compounds are formed, which are eventually converted into solid residues.
(5) Final Product—The carbonaceous residue (coke deposit) that remains after the completion of all degradation stages.

### 3.2. Investigation by IR Spectroscopy and Quantum Chemistry of p-PGFPh:AA

Infrared (IR) spectroscopy can effectively determine the presence of key functional groups in the copolymers based on polypropylene glycol fumarate phthalate, such as fumarate, phthalate, and acrylate groups, as well as hydroxyl groups. As part of the study, an attempt was made to modify the IR spectra for a more detailed analysis of the polymers’ structural features, which allowed for the identification of subtle changes in functional groups and interactions, thus contributing to a deeper understanding of its chemical properties and thermal degradation.

According to [32], the experimental IR spectrum (in transmittance intensity (%), wavenumber (cm^−1^)) of polypropylene glycol fumarate phthalate (p-PGFPh) shows absorption bands at 3535, 2981, and 2355 cm^−1^, as well as in the range of 1724–1730 cm^−1^:


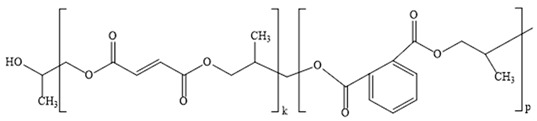


A comparative analysis of the results of quantum chemical calculations of the vibrational absorption spectrum of the model molecular structure and the experimental spectrum of p-PGFPh was carried out to assign all bands in the IR spectrum of p-PGFPh.

Quantum chemical calculations of the structure and properties of a model compound consisting of ethylene and phenylene-containing monomeric units:


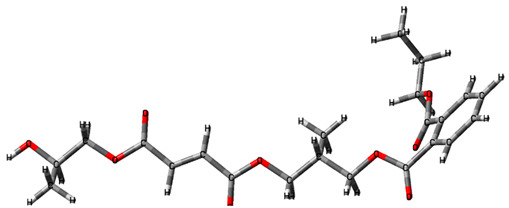


Additionally, the vibrational frequencies and intensities of the IR absorption bands for the model were performed using the density functional theory (DFT) method with the Becke 3 Lee Yang Parr (B3LYP) correlation functional and the valence split basis set 6-31G(*d*) (Gaussian 16 software package) [33].

**Figure 5:**
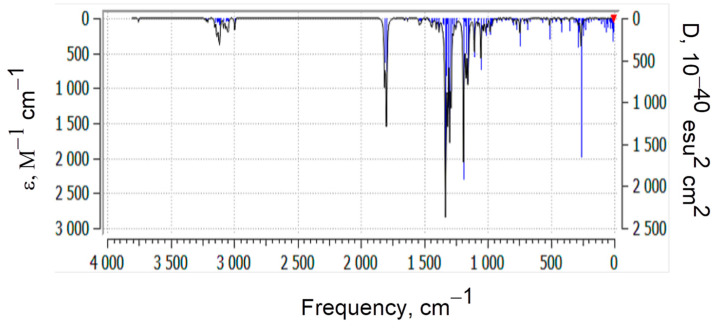
The vibrational spectrum of the model compound calculated using the B3LYP/6-31G(*d*) method.

Figure 5 presents the theoretical quantum chemical vibrational spectrum (in absorption coefficient vs. wavenumber, or frequency coordinates) of the model compound. The vibrational spectrum of the model molecule, consisting of 59 atoms, contains 171 normal modes of vibration. In the theoretically calculated spectrum, a fragile band appears at a frequency of 3753.35 cm^−1^ (it is known that theoretical frequencies are typically higher than experimental values), which corresponds to the valence vibration of the free hydroxyl group present in the model. In the experimental spectrum of p-PGFPh, a medium-intensity band is observed at 3534.76 cm^−1^, which, in our opinion, may correspond to the first overtone of carbonyl group absorption, or this frequency could be attributed to trace amounts of OH groups. Another absorption band in the experimental spectrum at ν_exp_ 2980.96 cm^−1^ likely belongs to the valence vibrations of C–H bonds in methylene and/or methyl, methine, or phenyl groups. In the theoretical spectrum of the model compound, the absorption region in the 3800-2900 cm^−1^ range corresponds to the asymmetric and symmetric vibrations of C–H bonds in these groups.

The spectrum of the model compound does not exhibit the band observed in the experimental FT-IR spectrum at ν_exp_ 2355.33 cm^−1^. This band occurs within the transparency region and is typically attributed to a phosphate group. However, since the polymer does not contain phosphorus, two possible origins for this band can be proposed: it may result from the valence vibrations of non-associated OH groups, or it could be due to hydrochloric acid salts of amines. The most intense band in the experimental IR spectrum (ν_exp_ 1724.12 cm^−1^) is due to the valence vibrations of the polar C=O bonds. Because of conjugation of the carbonyl groups with the ethylene and benzene ring bonds, this frequency is apparently lower than expected. On the other hand, this frequency is higher than the corresponding frequency for ketones (1715 cm^−1^), which confirms the well-known fact that the frequency of carbonyl group vibrations increases when the group is conjugated with an electron-accepting atom, such as oxygen. In the region of the double bond, sharp, low-intensity bands are observed around 1637 and 1450 cm^−1^, which may correspond to the vibrations of C=O and C=C groups, including aromatic carbon–carbon bonds.

**Figure 6:**
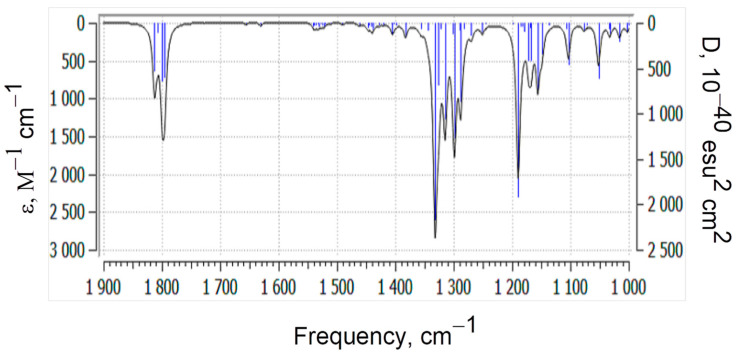
Absorption region 1900–1000 cm^−1^ of the theoretical spectrum of the model compound (B3LYP/6-31G(*d*) method.

Below is the theoretical absorption region of the model molecule from 1900 to 1000 cm^−1^ (Figure 6). According to the B3LYP/6-31G(*d*) data, in the range of 1813–1795 cm^−1^, very intense asymmetric and symmetric valence vibrations of C=O groups are observed. The order of the C=O bonds remains unchanged, as indicated by the bond lengths (0.122–0.125 nm). Very weak signals are observed at 1656 and 1631 cm^−1^, which belong to the valence vibrations, and at 1530–1532 and 1491 cm^−1^, which correspond to the deformation vibrations of the phenylene group. Valence vibrations of C–C bonds, changes in the valence angles of the C–O–C bonds, scissor vibrations of methylene groups, symmetric and asymmetric deformation vibrations of methyl groups, and deformation vibrations of the ring (rocking) are observed in the “fingerprint” region (below 1500 cm^−1^).

**Figure 7:**
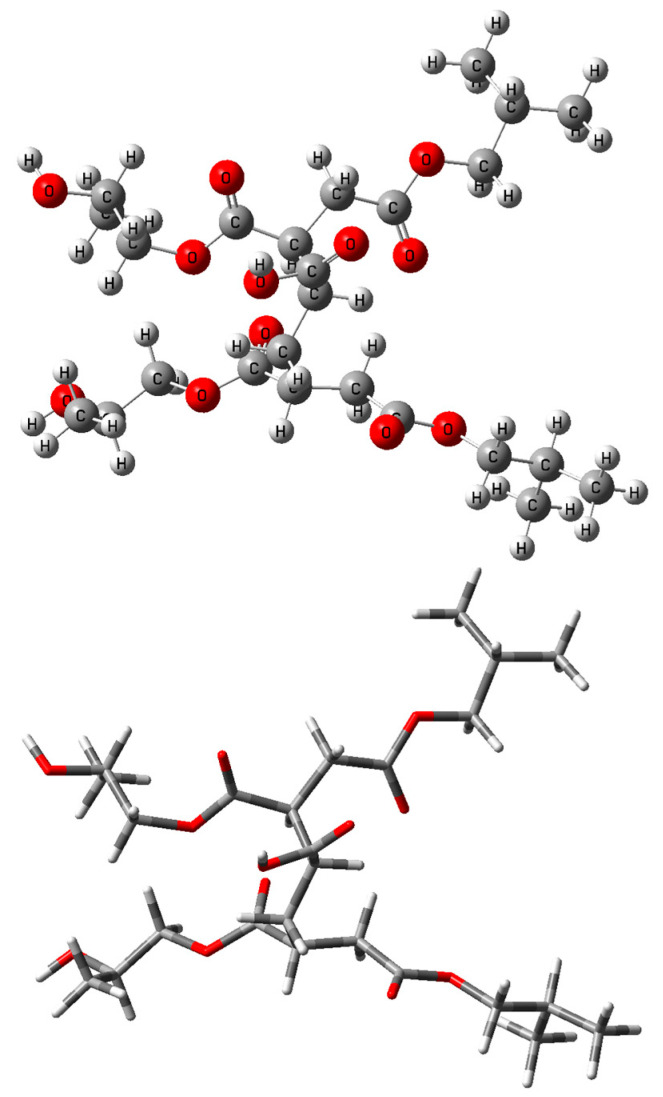
Model of the p-PGFPh copolymers with acrylic acid.

In the framework of this study, a comparative analysis of the results of quantum chemical calculations of the vibrational absorption spectrum of the model molecular structure (Figure 7) and the experimental spectrum of the p-PGFPh copolymers with AA was carried out for the identification of bands in the IR spectrum. Quantum chemical calculations of the structure and properties, as well as the vibrational frequencies and intensities of the IR absorption bands of the model structure, were performed using the density functional theory (DFT) method with the Becke 3 Lee Yang Parr (B3LYP) correlation functional and the valence split basis set 6-31G(*d*) (Gaussian 16 software package) [33].

**Figure 8:**
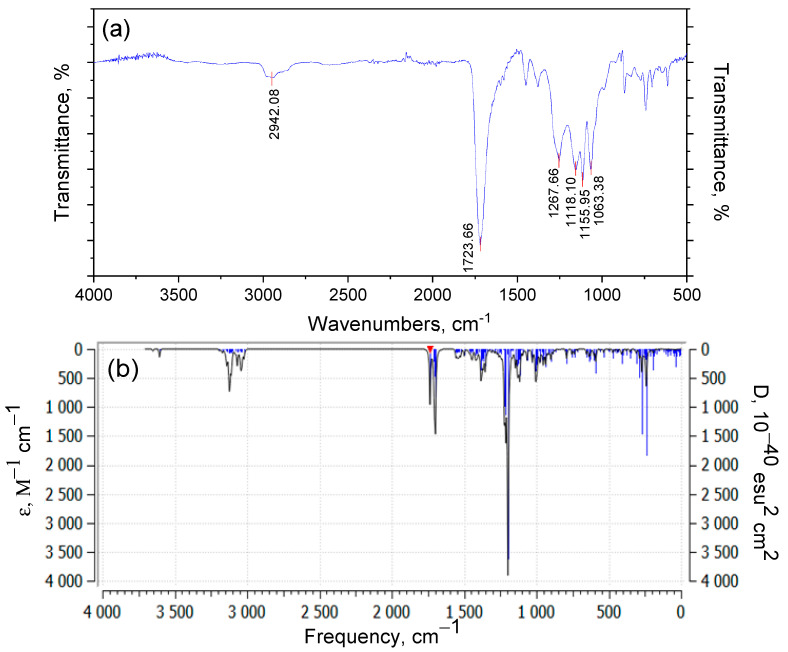
Experimental IR spectrum of the p-PGFPh:AA copolymers with acrylic acid (**a**) and calculated-theoretical vibrational spectrum of the model structure (**b**).

Figure 8 shows the experimental IR spectrum of the p-PGFPh copolymers with acrylic acid [32] and the theoretical quantum chemical vibrational spectrum of the model structure.

The vibrational spectrum of the model structure, consisting of 79 atoms, contains 231 normal modes of vibration. In the theoretically calculated spectrum, very weak bands are observed in the range of 3750–3600 cm^−1^ (it is known that theoretical frequencies are typically higher than experimental ones), corresponding to the valence vibrations of free hydroxyl groups present in the model. Very weak signals from these groups are likely observed near 4000 cm^−1^ in the experimental spectrum. The next frequency group in the theoretical spectrum corresponds to the absorption region of 3200–3000 cm^−1^ and relates to the asymmetric and symmetric valence vibrations of C–H bonds in methylene and/or methyl, as well as methine groups. The corresponding band in the experimental spectrum appears at 2942 cm^−1^.

The most intense band in the experimental IR spectrum (ν_exp_ 1723.66 cm^−1^) is due to the valence vibration of the C=O bond in the carboxyl group. In the frequency range of 1735–1700 cm^−1^ of the model spectrum, signals are attributed to the vibrations of polar bonds in the carbonyl groups and the carboxyl group, with very high intensity.

Valence vibrations of C–C bonds, changes in the valence angles of C–O–C bonds, scissor vibrations of methylene groups, and symmetric and asymmetric deformation vibrations of methyl groups are observed in the “fingerprint” region (below 1500 cm^−1^). In the experimental spectrum, absorption bands are present at frequencies of 1268, 1118, and 1063 cm^−1^, which are responsible for the aforementioned vibrations.

For clarity, Table 1 compares the key experimental absorption bands with the corresponding theoretical values and their vibrational assignments.

Table 1 demonstrates the correspondence between the experimental and calculated IR absorption bands of the p-PGFPh:AA copolymers, supporting the identification of structural fragments involved in thermal decomposition. For example, the band observed at 1724–1723 cm^−1^, which aligns with the calculated range of 1735–1700 cm^−1^, corresponds to the stretching vibrations of C=O groups, indicating the degradation of ester and carboxylic moieties. Vibrations in the region of 2980–2942 cm^−1^ (experimental) and 3020–2950 cm^−1^ (calculated) reflect C–H bonds in aliphatic and aromatic groups, which undergo cleavage during the initial stages of decomposition. Data on CH_2_ and CH_3_ deformation vibrations (1268–1063 cm^−1^) help to identify unstable segments of the polymer backbone.

Particular attention should be given to an unassigned band at 2355 cm^−1^, which is absent in the calculated spectrum. It is most likely attributed to atmospheric CO_2_ and unrelated to intrinsic polymer vibrations.

Thus, the table provides a basis for interpreting the mechanisms of thermal degradation and correlating them with TGA data.

Table 2 presents the kinetic parameters obtained from experimental and theoretical data for comparative analysis. The kinetic parameters were calculated using methods of linear algebra and numerical analysis, including Cholesky decomposition, normal equations, singular value decomposition (SVD), and QR decomposition. To assess the reliability of the results, the values of E_a_ and A are presented in the “mean ± standard deviation” format, based on three independent experiments. The low SD values confirm the reproducibility and robustness of the obtained data.

Kinetic analysis (Table 2) revealed that the p-PGFPh:AA copolymer with a composition of 6.77:93.23 exhibit higher activation energy (*E_a_* ≈ 216.13 kJ·mol^−1^), indicating enhanced thermal stability, particularly under oxidative (air) conditions. Although copolymers with a higher content of polypropylene glycol fumarate phthalate (PPGFP) show slightly lower activation energies (*E_a_* ≈ 204.18–207.25 kJ·mol^−1^) compared to acrylic acid-rich compositions, the incorporation of PPGFP as a monomer unit in hydrogel synthesis provides several important advantages. Specifically, the presence of hydrophobic phthalate and propylene glycol fragments enables more precise control over the water retention capacity, which is critical for developing hydrogels with delayed water or active substance release. Moreover, the unsaturated moieties in the PPGFP structure impart high reactivity in copolymerization reactions, allowing:

- Efficient integration of the monomer into a three-dimensional crosslinked polymer matrix;
- Fine-tuning of the composition for specific applications, including the grafting of biologically active fragments.

The resulting copolymers demonstrate slow but controllable thermal degradation, which is particularly advantageous for the development of temporary implants and controlled drug delivery systems. In addition, phthalate segments contribute to improved hydrolytic stability, especially in mildly acidic and microbiologically active environments such as soil or biological fluids, thereby expanding the application potential of PPGFP-based hydrogels in biomedical and environmental technologies. These findings imply that the experimentally determined degradation rates and mechanisms are directly linked to the durability and long-term performance of these materials under real-world conditions. For instance, resistance to decomposition in soil—where moisture and microorganisms are present—can be predicted based on the kinetic parameters obtained. Thus, the analysis confirms that the developed hydrogels are well suited for practical applications in agriculture, environmental remediation, and medicine. Despite a slight reduction in thermal stability, the structural and functional benefits of such copolymers are not only retained but, in many cases, enhanced. This supports their high potential for use in hydrogel systems designed to function under hydrolytic stress, microbial exposure, and elevated temperatures—such as soil conditioners, slow-release delivery systems, and implantable biomedical materials.

For each copolymer composition (6.77:93.23 and 86.67:13.33 mol%) in both nitrogen and air atmospheres, one-way analysis of variance (ANOVA) and pairwise *t*-tests were conducted. The results are included in Supplementary Materials S4.

**Figure 9:**
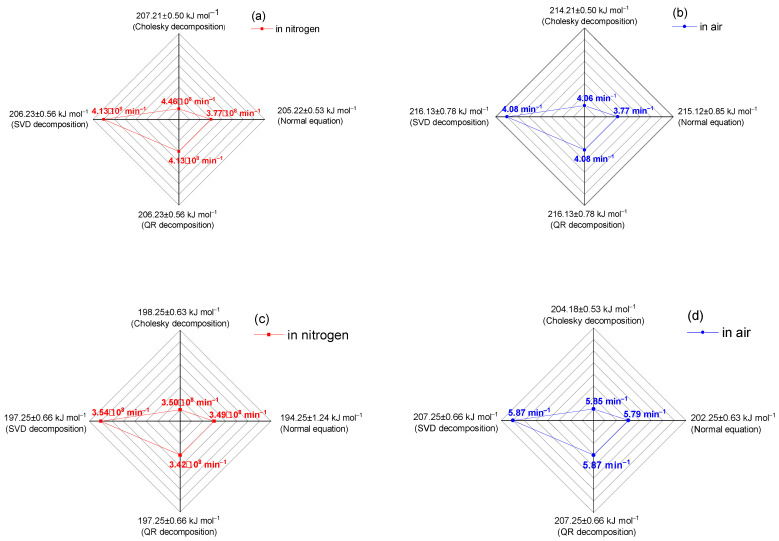
Activation energy and pre-exponential factor as a function of the partial peak area for the p-PGFPh:AA copolymers at initial M_1_:M_2_ ratios, mol.%: (**a**,**b**) 6.77:93.23 mol.%, (**c**,**d**) 86.67:13.33 mol.%.

Figure 9 shows the dependence of the activation energy (in kJ mol^−1^) and the pre-exponential factor (in min^−1^) as a function of the partial peak area for different conditions and calculation methods. The red lines in graphs (a) and (c) correspond to the nitrogen atmosphere, while the blue lines in graphs (b) and (d) correspond to the air atmosphere. The results in Figure 9 show that the decomposition method affects the value of the activation energy and the pre-exponential factor, with QR decomposition and SVD decomposition improving resistance to rounding errors. This ensures the accuracy and stability of these methods in calculating dynamic parameters.

## 4. Conclusions

This study presents a detailed analysis of the thermal degradation behavior of copolymers based on polypropylene glycol fumarate phthalate (p-PGFPh) and acrylic acid. Using polynomial approximation, linear algebra methods, and quantum chemical calculations, the degradation stages were analyzed and the kinetic parameters were estimated. The results revealed that structural characteristics such as the acrylic acid content and the nature of ether and ester linkages significantly affect the onset temperature and degradation mechanism. The integration of TGA data with quantum chemical modeling enabled the identification of thermolabile molecular fragments and the prediction of thermal stability trends. These findings may be utilized for the design of more thermally stable copolymeric materials through the optimization of monomer ratios, increased crosslinking density, or the incorporation of stabilizing groups into the polymer backbone. This is particularly relevant for applications in agriculture (e.g., water-retaining hydrogels) and environmental protection (e.g., materials for wastewater treatment). Future research will focus on experimental validation of the degradation pathways under various external conditions (e.g., humidity, UV radiation, and oxygen exposure), as well as the extension of the theoretical framework using more advanced quantum chemical approaches, such as time-dependent DFT (TD-DFT) and ab initio molecular dynamics (AIMD). Further kinetic analysis of copolymers containing alternative functional comonomers could also contribute to the development of novel smart polymer systems.

Mathematical models were used to describe the thermograms of the p-PGFPh:AA copolymers, which accurately approximated the experimental data and revealed the main regularities of mass change in the material as a function of temperature. The experimental data were processed using a polynomial model, such as the Poly ($y=a_{0}+a_{1}x+a_{2}x^{2}+\ldots +a_{n-1}x^{n-1}$) function, which significantly improved the accuracy of the results. This approach not only enhanced the quality of data fitting but also made deeper analysis possible. All corrected *R^2^* values exceeded 0.99, indicating a high degree of agreement with the experimental data. 

The study discusses various quantum chemistry methods, such as the density functional theory (DFT) with the Becke 3 Lee Yang Parr (B3LYP) correlation functional and the valence split basis set 6-31G(*d*), which are used to calculate the characteristics of molecules based on polypropylene glycol fumarate phthalate (p-PGFPh). The main approaches to the calculation of vibrational frequencies, including the construction of information matrices and solving the force constant equations, are also discussed. The advantages and disadvantages of each method are analyzed, allowing for the selection of the optimal approach for specific tasks. 

In the study of polymer degradation based on polypropylene glycol fumarate phthalate, it is demonstrated that various computational methods based on the least squares method have specific advantages and disadvantages that affect the accuracy of the calculations. The analysis presented in Table 2 shows that most methods, except for direct estimation, provide high accuracy for both the activation energy and pre-exponential factor. Interestingly, the results obtained using normal equations and QR decomposition methods coincide up to the fourth decimal place, indicating their close interrelationship. This is because QR decomposition, using the Gram–Schmidt orthogonalization procedure, preserves the original data values, which is an important aspect in calculations based on the radial distribution function (RDF). However, QR decomposition demonstrates a significant advantage in terms of resistance to rounding errors, making it the preferred choice in situations where data accuracy is critical. By contrast, in the absence of rounding issues, the normal equations method may be more feasible as it requires less computational effort. Thus, when computation speed is the highest priority, normal equations and Cholesky factorization methods are more advantageous, as their application allows for much faster solutions, especially when dealing with large datasets. Singular value decomposition (SVD) is still difficult to compute but remains the only method applied when *T* has incomplete rank. These methods are highly valuable, both in terms of the information they provide and the speed and flexibility of their calculations.

## 5. Patents

The methods for preparing the unsaturated polyester resins described in this manuscript, including the use of polypropylene glycol fumarate phthalate as a key component, are protected by a patent [15].

## Figures and Tables

**Table 1:** Comparison of experimental and calculated IR wavenumbers and their vibrational assignments.

Wavenumber of Experimental Peak (cm^−1^)	Calculated Wavenumber (cm^−1^)	Vibrational Assignment
3535	3753	Stretching vibrations of free OH groups
2980–2942	3020–2950	C–H stretching vibrations of CH_3_, CH_2_, and phenyl groups
2355	—	Atmospheric CO_2_ or trace impurities
1724–1723	1735–1700	C=O stretching modes in esters and carboxylic acids
1637, 1450	1656, 1491	Aromatic and C=C deformation modes
1268, 1118, 1063	1270–1050	CH_2_ and CH_3_ deformation vibrations, and ring mode vibrations

**Table 2:** Kinetic parameters of the thermodegradation process of the p-PGFPh:AA copolymers. Note: Activation energy (*E_a_*) is given in kJ mol^−1^; pre-exponential factor (*A*) is given in min^−1^. All values are presented as mean ± standard deviation from triplicate measurements.

Sample	Cholesky Decomposition		Normal Equations		Singular Value Decomposition		QR Decomposition	
	E_a_ (kJ mol^−1^) ±SD	A·10^8^, (min^−1^) ±SD	E_a_ (kJ mol^−1^) ±SD	A·10^8^, (min^−1^) ±SD	E_a_ (kJ mol^−1^) ±SD	A·10^8^, (min^−1^) ±SD	E_a_ (kJ mol^−1^) ±SD	A·10^8^, (min^−1^) ±SD
EXPERIMENTAL DATA								
Nitrogen								
6.77:93.23	207.21 ±0.50	4.46 ±0.26	205.22 ±0.53	3.77 ±0.30	206.23 ±0.56	4.13 ±0.13	206.23 ±0.56	4.13 ±0.13
86.67:13.33	198.25 ±0.63	3.50 ±0.50	194.25 ±1.24	3.49 ±0.38	197.25 ±0.66	3.54 ±0.10	197.25 ±0.66	3.42 ±0.13
Air								
6.77:93.23	214.21 ±0.50	4.06 ±0.29	215.12 ±0.85	3.77 ±0.25	216.13 ±0.78	4.08 ±0.05	216.13 ±0.78	4.08 ±0.05
86.67:13.33	204.18 ±0.53	5.85 ±0.15	202.25 ±0.63	5.79 ±0.30	207.25 ±0.66	5.87 ±0.05	207.25 ±0.66	5.87 ±0.05
THEORETICAL DATA								
6.77:93.23	196.98 ±0.25	1.90 ±0.34	194.98 ±0.25	1.90 ±0.34	195.98 ±0.25	1.90 ±0.34	195.98 ±0.25	1.90 ±0.34
86.67:13.33	171.20 ±0.47	4.83 ±0.75	170.20 ±0.47	4.83 ±0.75	170.17 ±0.47	4.83 ±0.75	170.20 ±0.47	4.83 ±0.75

## Data Availability

The original contributions presented in this study are included in the article/supplementary material. Further inquiries can be directed to the corresponding author.

## Supplementary Materials

The following supporting information can be downloaded at: https://www.mdpi.com/article/10.3390/polym17091197/s1, Supplementary Materials S1: Conducting Experiments; Supplementary Materials S2: Python Code for Kinetic Analysis; Supplementary Materials S3: Thermogravimetric analysis (TGA) curves. (Figure S1: Thermogravimetric analysis (TGA) curves, derivatives of thermogravimetry (DTG), and elemental composition of p-PGFPh:AA copolymers under an air atmosphere: (a,c) 6.77:93.23 and (b,d) 86.67:13.33); Supplementary Materials S4: Comparative Statistical Analysis of E_a_ Estimates; Supplementary Materials S5: Synthesis of p-PGFPh. (Figure S2. ^1^H NMR spectrum of p-PGFPh in CDCl_3_ solution; Figure S3. ^13^C NMR spectrum of p-PGFPh in CDCl_3_ solution); Supplementary Materials S6: Synthesis of p-PGFPh. Synthesis of Copolymers of p-PGFPh.
