# Supplementary material for: Study of Mathematical Models Describing the Thermal Decomposition of Polymers Using Numerical Methods
Source: Polymers (Basel). 2025 Apr 27;17(9):1197. doi: 10.3390/polym17091197 (PMC12073326; doi:10.3390/polym17091197)
Supplement: Supplementary file 1 [file polymers-17-01197-s001.zip › Supplementary Materials_1.pdf]

Supplementary Materials S1

Conducting Experiments

To determine the thermodynamic parameters of the synthesized copolymers, thermogravimetric measurements (TGA-DSC-IR and TGA-DSC-MS) were performed, recording curves on the Netzsch Jupiter STA 449 F3 analyzer, combined with a Bruker FTIR alpha II IR and mass spectrometer. Samples (30 mg, powdered form) were heated in the temperature range of 50–800°C at a rate of 10.0°C/min under a nitrogen atmosphere. To increase the accuracy of the measurements, each experiment was conducted three times, after which the average values were calculated to minimize random errors and improve data reproducibility.

Calibration of the thermogravimetric analyzer was performed using certified metal standards (indium, tin, aluminum) in accordance with the procedures outlined in ASTM E1582-00: Standard Practice for Calibration of Temperature Scale for Thermogravimetry. This ensured the accuracy of the temperature readings during the decomposition analysis.

References

[1] ASTM E1582-00 (Reapproved 2016), Standard Practice for Calibration of Temperature Scale for Thermogravimetry, ASTM International, West Conshohocken, PA, 2016. https://www.materials.co.uk/tga.htm
