# Supplementary material for: Study of Mathematical Models Describing the Thermal Decomposition of Polymers Using Numerical Methods
Source: Polymers (Basel). 2025 Apr 27;17(9):1197. doi: 10.3390/polym17091197 (PMC12073326; doi:10.3390/polym17091197)

Supplementary Materials S2

Below is the code in the Python programming language that implements this algorithm. The code uses the numpy library for working with arrays and vectors, and pandas for convenient handling of csv files in the Jupyter notebook environment.

In [1]:

```
import numpy as np
import pandas as pd

data=pd.read_csv("C:\\Users\\MPAES\\Desktop\\data.csv",sep=',',names=['T','h'],index_col=
None)
xx=data['T']
yy=data['h']
def coefficient_reg(x,y):
size=len(x)
# we form and fill the matrix of size 2x2
A=np.empty((2,2))
A[[0],[0]]=sum((x[i])**2 for in range(0,size))
A[[0],[1]]=sum(x)
A[[1],[0]]=sum(x)
A[[1],[1]]=size
# we find the inverse matrix
A=np.linalg.inv(A)
# we form and fill the matrix of dimensions 2x1
C=np.empty((2,1))
C[0]=sum((x[i]*y[i]) for in range(0,size))
C[1]=sum((y[i]) for in range(0,size))
# we multiply the matrix by the vector
ww=np.dot(A,C)
return ww[1], ww[0]

[w0_1,w1_1]=coefficient_reg(xx,yy)
print(w0_1,w1_1)
```

Out [1]:

```
[19.53799407] [-10.65148089]
```

You can also use the sklearn library for data analysis and machine learning, specifically the LinearRegression class.

In [2]:

```
from sklearn.linear_model import LinearRegression
from sklearn.model_selection import train_test_split
# we find the coefficients of the linear regression
X_train,X_test,y_train,y_test=train_test_split(data['T'],data['h'],random_state=42)
lr=LinearRegression()
lr.fit(X_train.to_frame(name='T'),y_train)
print("lr.coef_: {}".format(lr.coef_))
print("lr.intercept_: {}".format(lr.intercept_))
```

Out [2]:

```
lr.coef_: [-10.40173639]
lr.intercept_: 19.11816463123632
```

We see that the coefficients found by two different methods match. To check how well our linear model describes the data, we plotted graphs with the data and the data obtained using the linear model (lr.coef_ = -10.40173639, lr.intercept_ = 19.11816463123632). For visualization, we used the library Matplotlib. The code for visualizing the graphs is provided below:

In [3]:

```
import matplotlib.pyplot as plt
plt.plot(data['T'],data['h'],'o')
x=np.linspace(data['T'].min(),data['T'].max(),100)
y=lr.intercept_+lr.coef_*x
plt.plot(x,y)
```

Out[3]:

```
[<matplotlib.lines.Line2D at 0x17bf2abea50>]
```

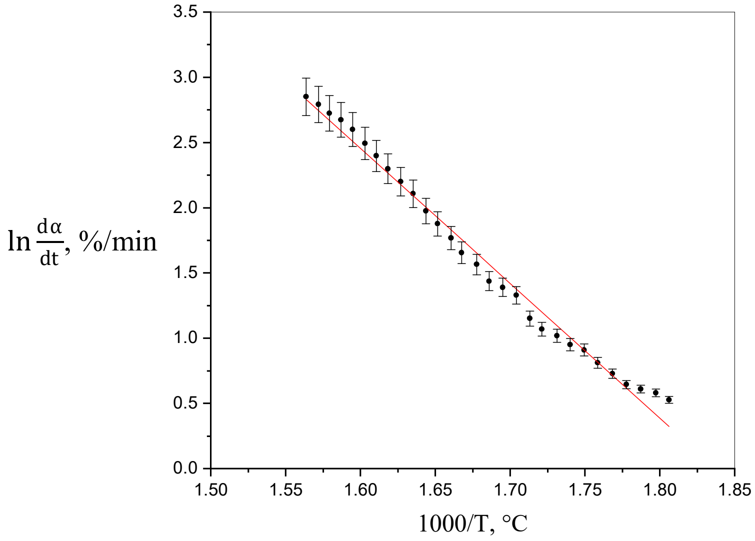

Supplement: Supplementary file 1 [file polymers-17-01197-s001.zip › Supplementary Materials_2.pdf]
