# Supplementary material for: Study of Mathematical Models Describing the Thermal Decomposition of Polymers Using Numerical Methods
Source: Polymers (Basel). 2025 Apr 27;17(9):1197. doi: 10.3390/polym17091197 (PMC12073326; doi:10.3390/polym17091197)
Supplement: Supplementary file 1 [file polymers-17-01197-s001.zip › Supplementary Materials_3.pdf]

Supplementary Materials S3

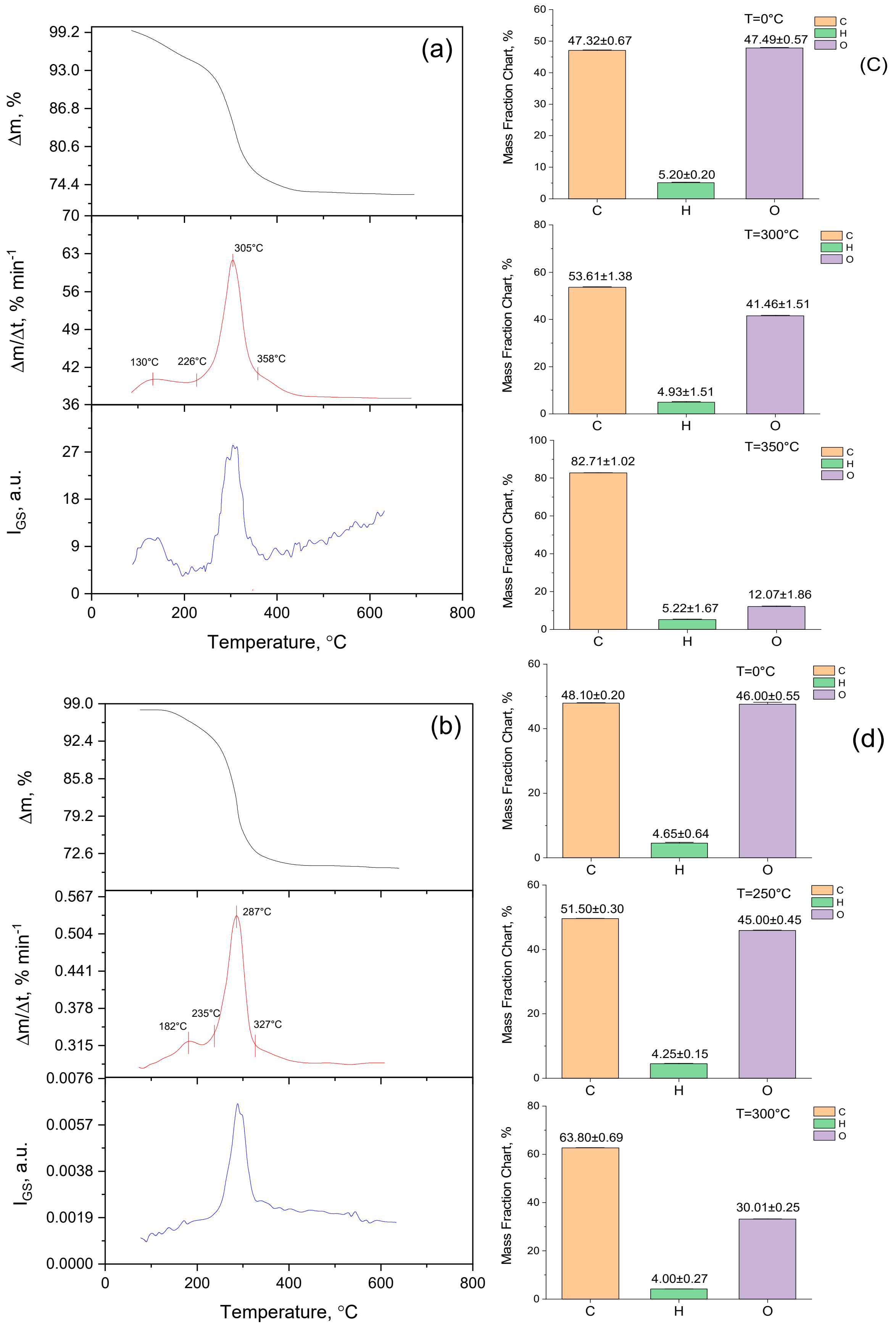

Figure S1. Thermogravimetric analysis (TGA) curves, derivatives of thermogravimetry (DTG), and elemental composition of p-PGFPh:AA copolymers under an air atmosphere: (a, c) 6.77:93.23 and (b, d) 86.67:13.33.
