# Supplementary material for: Study of Mathematical Models Describing the Thermal Decomposition of Polymers Using Numerical Methods
Source: Polymers (Basel). 2025 Apr 27;17(9):1197. doi: 10.3390/polym17091197 (PMC12073326; doi:10.3390/polym17091197)
Supplement: Supplementary file 1 [file polymers-17-01197-s001.zip › Supplementary Materials_4.pdf]

Supplementary Materials S4

Comparative Statistical Analysis of Ea Estimates

To assess the statistical significance of differences in activation energy (Ea) values calculated using various numerical methods (Cholesky decomposition, normal equations, SVD, and QR decomposition), one-way analysis of variance (ANOVA) and pairwise t-tests were performed. The analysis was conducted separately for two copolymer compositions 6.77:93.23 and 86.67:13.33 (mol%), under both nitrogen and air atmospheres.

ANOVA Results

Composition	F-statistic	p-value
Nitrogen		
6.77:93.23	24.20	9.43×10^{-9}
86.67:13.33	30.98	4.45×10^{-10}
Air		
6.77:93.23	301.2	1.34×10^{-25}
86.67:13.33	130.6	1.78×10^{-21}
Theoretical data		
6.77:93.23	52.61	1.03×10^{-8}
86.67:13.33	6.36	0.0025

Pairwise t-Test: Composition 6.77:93.23 (Nitrogen)

Comparison	p-value	Interpretation of Results
Cholesky vs Normal Eq.	< 0.001	Significant difference
Cholesky vs SVD	0.0002	Significant difference
Cholesky vs QR	0.0004	Significant difference
Normal Eq. vs SVD	0.0003	Significant difference
Normal Eq. vs QR	0.0036	Significant difference
SVD vs QR	0.96	Not significant (results similar)

Pairwise t-Test: Composition 86.67:13.33 (Nitrogen)

Comparison	p-value	Interpretation
Cholesky vs Normal Eq.	< 0.001	Significant difference
Cholesky vs SVD	0.02	Significant difference
Cholesky vs QR	0.02	Significant difference
Normal Eq. vs SVD	< 0.001	Significant difference
Normal Eq. vs QR	< 0.001	Significant difference

SVD vs QR	0.99	Not significant (results similar)

Pairwise t-Test: 6.77:93.23 (Air)

Comparison	p-value	Interpretation
Cholesky vs Normal Eq	1.29×10^{-4}	Significant difference
Cholesky vs SVD	1.78×10^{-11}	Significant difference
Cholesky vs QR	2.03×10^{-11}	Significant difference
Normal Eq vs SVD	6.99×10^{-12}	Significant difference
Normal Eq vs QR	7.99×10^{-12}	Significant difference
SVD vs QR	0.99	Not significant (results similar)

Pairwise t-Test: 86.67:13.33 (Air)

Comparison	p-value	Interpretation
Cholesky vs Normal Eq	< 0.001	Significant difference
Cholesky vs SVD	< 0.001	Significant difference
Cholesky vs QR	< 0.001	Significant difference
Normal Eq vs SVD	< 0.001	Significant difference
Normal Eq vs QR	< 0.001	Significant difference
SVD vs QR	≈1.0	Not significant (results similar)

Pairwise t-Test t- Test: 6.77:93.23 (theoretical data)

Comparison	p-value	Interpretation
Cholesky vs Normal Eq.	0.000012	Significant difference
Cholesky vs SVD	0.000921	Significant difference
Cholesky vs QR	0.000072	Significant difference
Normal Eq. vs SVD	0.265	Significant difference
Normal Eq. vs QR	0.158	Significant difference
SVD vs QR	≈1.0	Consistent results

Pairwise t-Test t- Test: 86.67:13.33 (theoretical data)

Comparison	p-value	Interpretation
Cholesky vs Normal Eq.	< 0.001	Significant difference
Cholesky vs SVD	< 0.001	Significant difference
Cholesky vs QR	< 0.001	Significant difference
Normal Eq. vs SVD	≈1.0	Consistent results
Normal Eq. vs QR	≈1.0	Consistent results
SVD vs QR	≈1.0	Consistent results
