# Supplementary material for: Study of Mathematical Models Describing the Thermal Decomposition of Polymers Using Numerical Methods
Source: Polymers (Basel). 2025 Apr 27;17(9):1197. doi: 10.3390/polym17091197 (PMC12073326; doi:10.3390/polym17091197)
Supplement: Supplementary file 1 [file polymers-17-01197-s001.zip › Supplementary Materials_5.pdf]

Supplementary Materials S5

Synthesis of p-PGFPh

Polypropylene glycol fumarate phthalate was obtained through the polycondensation reaction of propylene glycol, phthalic anhydride, and fumaric acid at a temperature of 453–473 K. The polycondensation was carried out according to standard procedures in the presence of an aluminum chloride catalyst under nitrogen in order to avoid undesirable gelatinization processes. The polycondensation was carried out for 16 hours.

I)

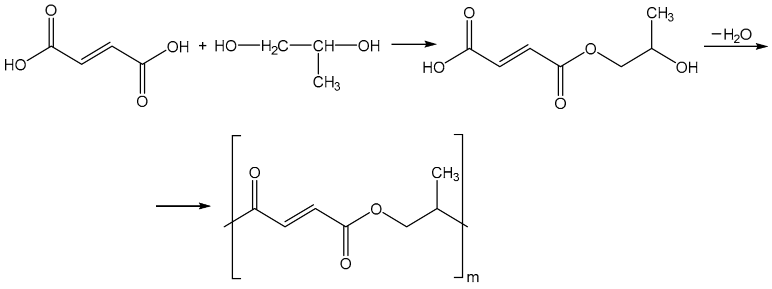

II)

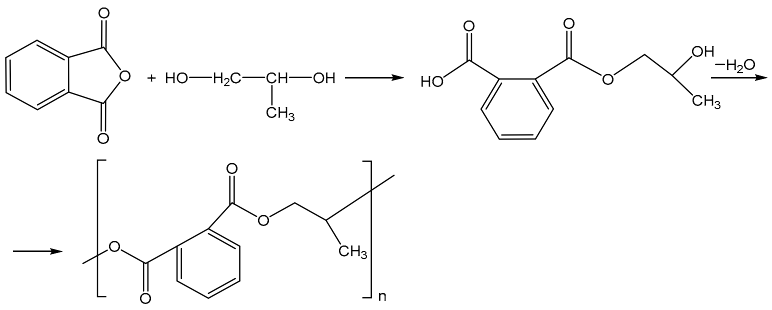

III)

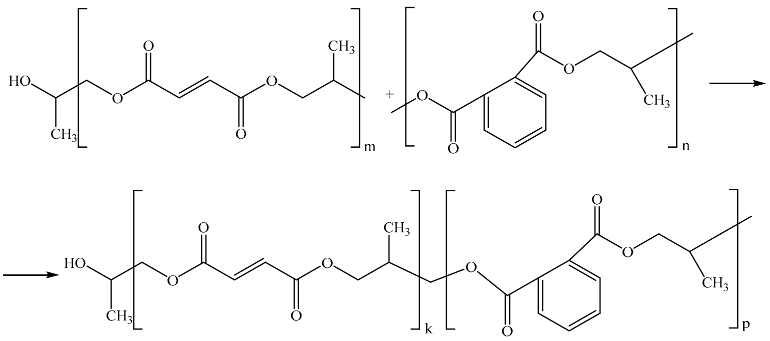

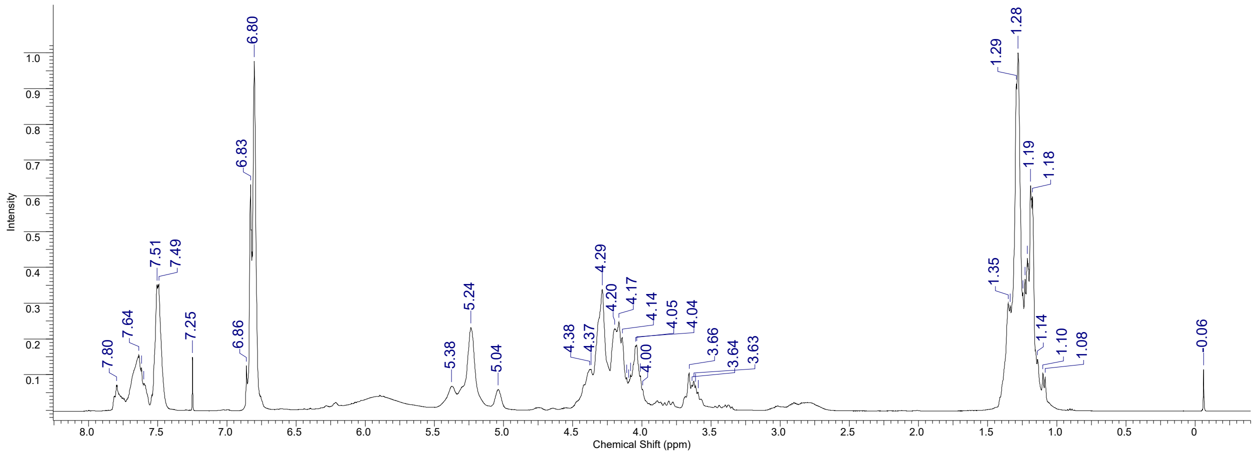

No.	(ppm)	(Hz)	Height	No.	(ppm)	(Hz)	Height	No.	(ppm)	(Hz)	Height	No.	(ppm)	(Hz)	Height
1	-0.06	-24.3	0.1145	13	1.35	540.8	0.3001	25	4.11	1643.5	0.0930	37	6.83	2730.3	0.6322
2	1.08	433.6	0.0937	14	3.59	1437.0	0.0502	26	4.14	1655.9	0.2045	38	6.86	2742.6	0.1255
3	1.10	440.1	0.1038	15	3.61	1443.9	0.0681	27	4.17	1666.0	0.2473	39	7.25	2898.3	0.1495
4	1.14	455.6	0.1428	16	3.63	1449.4	0.0816	28	4.19	1676.5	0.2271	40	7.49	2995.9	0.3515
5	1.18	470.3	0.5980	17	3.64	1454.4	0.0712	29	4.20	1678.3	0.2276	41	7.51	3000.9	0.3517
6	1.19	476.2	0.6282	18	3.66	1462.7	0.1046	30	4.29	1713.6	0.3379	42	7.60	3038.9	0.0757
7	1.21	484.9	0.4258	19	4.00	1597.3	0.0582	31	4.37	1746.6	0.1149	43	7.62	3046.2	0.1185
8	1.23	491.8	0.3662	20	4.01	1604.2	0.0970	32	4.38	1750.2	0.1146	44	7.64	3053.6	0.1557
9	1.25	498.2	0.3289	21	4.04	1614.2	0.1824	33	5.04	2015.4	0.0589	45	7.79	3116.3	0.0704
10	1.28	512.0	1.0000	22	4.05	1617.4	0.1807	34	5.24	2093.7	0.2313	46	7.80	3118.1	0.0710
11	1.29	517.0	0.9133	23	4.08	1632.1	0.0988	35	5.38	2149.1	0.0673				
12	1.34	534.4	0.2922	24	4.10	1638.0	0.0800	36	6.80	2719.7	0.9765				

Figure S2. ^{1}H NMR spectrum of **p-PGFPh** in $CDCl_3$ solution

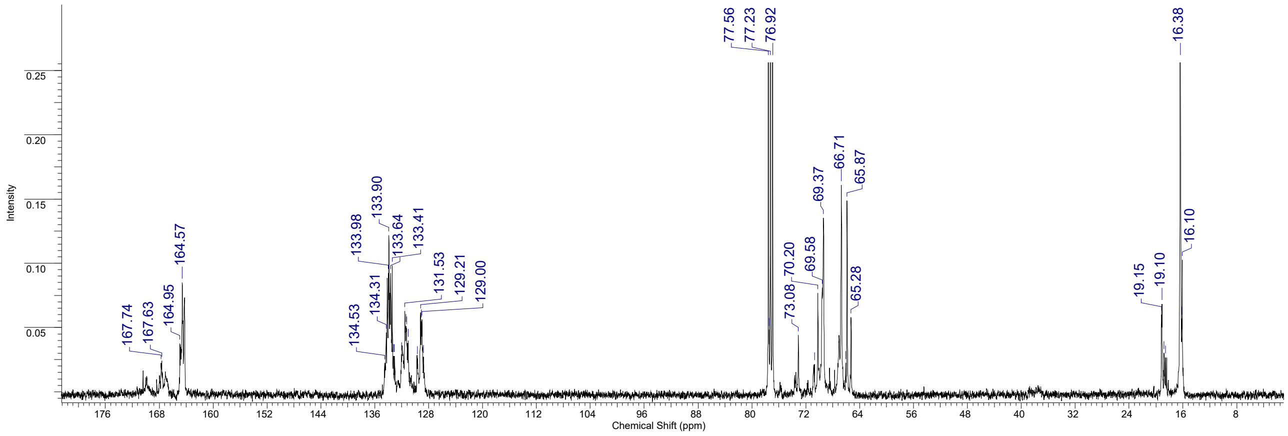

No.	(ppm)	(Hz)	Height	No.	(ppm)	(Hz)	Height	No.	(ppm)	(Hz)	Height	No.	(ppm)	(Hz)	Height
1	16.10	1618.0	0.1027	14	69.37	6973.0	0.1352	27	129.21	12989.3	0.0616	40	133.76	13446.5	0.0771
2	16.17	1625.7	0.0570	15	69.58	6995.0	0.0808	28	129.63	13031.4	0.0262	41	133.90	13459.9	0.1217
3	16.38	1646.8	0.2757	16	70.20	7057.3	0.0768	29	131.04	13173.3	0.0396	42	133.98	13468.5	0.0924
4	18.42	1851.9	0.0265	17	70.70	7107.2	0.0215	30	131.13	13181.9	0.0378	43	134.13	13483.8	0.0884
5	18.58	1868.2	0.0266	18	73.08	7346.8	0.0439	31	131.28	13197.3	0.0494	44	134.31	13501.1	0.0461
6	18.78	1888.3	0.0303	19	76.92	7732.1	0.9492	32	131.36	13204.9	0.0512	45	134.53	13524.1	0.0222
7	19.10	1919.9	0.0683	20	77.23	7763.7	1.0000	33	131.53	13222.2	0.0629	46	164.57	16543.3	0.0848
8	19.15	1924.7	0.0599	21	77.43	7783.8	0.0482	34	132.04	13273.0	0.0294	47	164.84	16570.1	0.0350
9	19.19	1929.5	0.0508	22	77.56	7796.3	0.9281	35	133.08	13377.5	0.0279	48	164.90	16576.8	0.0367
10	65.28	6561.8	0.0579	23	128.76	12943.3	0.0273	36	133.24	13393.7	0.0280	49	164.95	16581.6	0.0376
11	65.87	6621.2	0.1488	24	128.89	12956.7	0.0341	37	133.41	13411.0	0.0981	50	167.63	16850.9	0.0240
12	66.08	6642.3	0.0225	25	129.00	12968.2	0.0563	38	133.64	13434.0	0.0921	51	167.74	16862.4	0.0223
13	66.71	6705.6	0.1608	26	129.08	12975.9	0.0548	39	133.72	13442.6	0.0737				

Figure S3. ^{13}C NMR spectrum of **p-PGFPh** in $CDCl_3$ solution
