# Supplementary material for: Study of Mathematical Models Describing the Thermal Decomposition of Polymers Using Numerical Methods
Source: Polymers (Basel). 2025 Apr 27;17(9):1197. doi: 10.3390/polym17091197 (PMC12073326; doi:10.3390/polym17091197)

Supplementary Materials S6

Synthesis of p-PGFPh

Polypropylene glycol fumarate phthalate was obtained through the polycondensation reaction of propylene glycol, phthalic anhydride, and fumaric acid at a temperature of 453–473 K. The polycondensation was carried out for 16 h.

Synthesis of Copolymers of p-PGFPh

The radical copolymerization of p-PGFPh with AA was carried out in dioxane solution (1:1 by mass) in the presence of benzoyl peroxide (BP) as an initiating agent at 333 K. The ampoules were purged with an inert gas (N_2) for 30 min. Synthesis was carried out for 52 h. The resulting copolymer was washed with dioxane and dried to a constant weight in a vacuum oven.

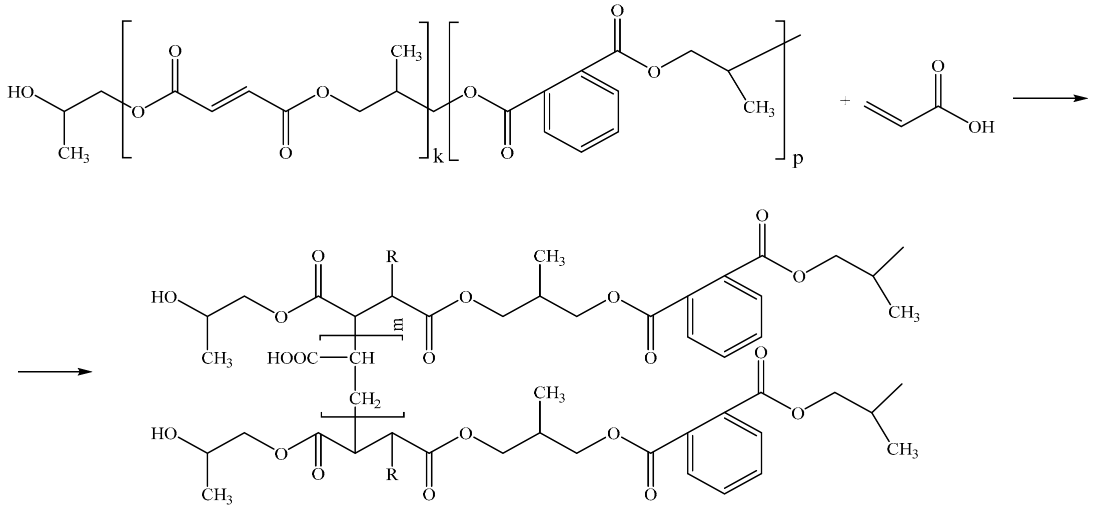

Supplement: Supplementary file 1 [file polymers-17-01197-s001.zip › Supplementary Materials_6.pdf]
